# Plasmon-Enhanced Asymmetry in the Charge Distribution Explains the Increased H_2_ Production Rate from Formic Acid with a Pd-Tipped Au Nanorod

**DOI:** 10.1021/acs.jpclett.5c02858

**Published:** 2025-12-11

**Authors:** Leonardo Biancorosso, Emanuele Coccia

**Affiliations:** Dipartimento di Scienze Chimiche e Farmaceutiche, 9315 University of Trieste, via L. Giorgieri 1, 34127, Trieste, Italy

## Abstract

Plasmonic nanostructures offer a promising route to increase efficiency in photocatalysis. This study provides a microscopic explanation for the enhanced H_2_ production rate from formic acid under plasmonic-resonance conditions of the photocatalytic reaction on a Pd-tipped Au nanorod (NR), observed experimentally. Using electron-dynamics multiscale simulations for a system composed of a classical Au NR and a DFT-described subsystem of Pd atoms and adsorbed reaction intermediates (bidentate HCOO* and H*) in the presence of a femtosecond pulse, we observe a net electron injection into HCOO*, which takes on the highest value in plasmon-resonance conditions. We find an asymmetry in the injection of electronic charge into the two oxygen atoms even in the absence of NR. The plasmonic field in resonant conditions significantly increases this asymmetry, thus representing the key to understanding the greater efficiency in H_2_ generation, since the next reaction step is the formation of the monodentate HCOO*. Also, a greater spatial heterogeneity of the charge on the Pd surface has been found in the case of resonance with the NR plasmon, which can promote the advancement of the reactive process.

## Graphical abstract

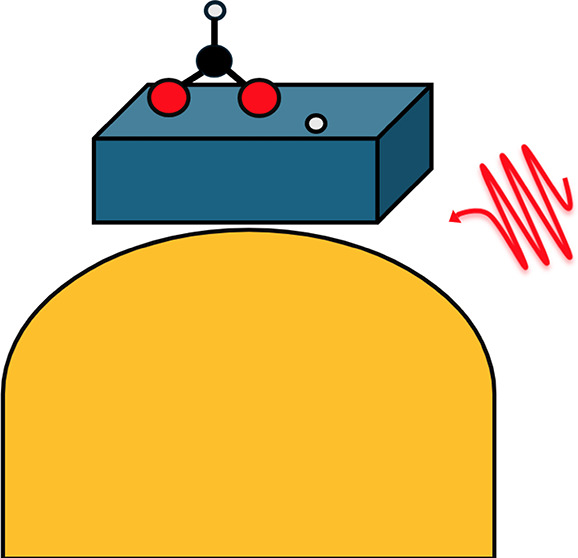

The search for alternatives to fossil fuel-based technologies has been one of the most prominent areas of research in recent decades.^1–3^ One of the most compelling directions in this area involves hydrogen production and storage, which holds great promise for clean energy applications.^4–9^ Plasmonic materials have successfully been exploited in photocatalysis through the concept of antenna–reactor complex, which has gained significant interest due to encouraging experimental and theoretical results.^10–16^ This hybrid system, which combines the light-harvesting properties of plasmonic systems and the catalytic features of metals such as Pd and Pt, offers a powerful platform for light-assisted chemical transformations thereby opening new avenues for plasmon-assisted catalysis.^17–27^

In the context of hydrogen generation, formic acid has been extensively used as hydrogen-rich compound.^28^ Herran et al. investigated various nanostructured configurations of palladium and Au, such as core–shell configurations and antenna–reactor assemblies, where small Pd particles are dispersed on the surface of a larger Au nanoparticle.^15^ They observed a pronounced boost in H_2_ production when the system was exposed to light, observing the best performance from antenna-reactor assemblies. Zheng and et al.^13^ have highlighted the role of localized surface plasmon resonances (LSPRs) in the catalytic enhancement of this reaction. In that work, the authors employed a tipped Au nanorod (NR) to investigate the decomposition of formic acid in H_2_. They observed a production rate of molecular hydrogen typically achieved at high temperature in thermal catalysis, highlighting a clear role of the plasmonic excitation of the NR.

By applying the same computational strategy recently adapted by some of us to explain the enhanced selectivity toward methane against carbon monoxide in the photocatalytic reduction of carbon dioxide in the presence of rhodium nanocubes,^10,29^ in this work we provide a microscopic explanation of the enhanced H_2_ production rate from formic acid in plasmonic-resonance conditions of the photocatalytic process on the tipped Au NR of ref 13. The analysis is based on the photo- and plasmon-induced charge injection into the stable reaction intermediate, i.e. the HCOO* moiety adsorbed with both oxygen atoms on the Pd surface.^30–38^ According to minimum-energy path calculations,^30,32^ the next step in the reaction is the formation of the monodentate HCOO* with only one oxygen adsorbed, which then leads to H_2_ and CO_2_. The key experimental quantity is the H_2_ production rate,^13^ which is enhanced in the presence of the gold NR and maximized at the NR plasmon frequency. In ref 13, the authors focus on the plasmonic near field to explain the observation without, however, proposing an atomistic description of the plasmon-mediated process. The idea behind our work is providing a direct link between the observed increase in H_2_ production and the plasmonic effects that modify the charge injection in the bidentate HCOO*, thus making the reaction more efficient toward the products.

The antenna-reactor complex plus HCOO* and H* is modeled as the following: the Au NR is treated by means of the polarizable continuum model (PCM), the reactor is given by two 2 × 3 layers of Pd atoms (2L3, details are provided in ref 38), and HCOO* and H* are adsorbed on the outer Pd layer. An explicit first-principle (QM) representation of the electronic degrees of freedom of Pd atoms, HCOO* and H* is given. The simulated Au NR has a radius of 3 nm and a length of 13.2 nm. It has the same aspect ratio of the reference NR in ref 13. A sketch representation of the simulated system is found in Figure 1. The theoretical framework is formulated in time domain using the time-dependent Schrödinger Equation (TDSE, eqs. 1–3 of the ) and the PCM model as well (TD-PCM-NP). The wavefunction of QM subsystem, i.e., Pd atoms + HCOO* + H*, |Ψ­(*t*)⟩ (eq. 4 of ) is defined in terms of the eigenstates of an effective Hamiltonian which contains the field-free Hamiltonian and the ground-state polarization of the NR.^39^ The NR surface is discretized in a set of *N*_*T*_ triangular tesserae, on which apparent charges are located.^40^ The time evolution of these charges allows us to describe the polarization of the NR according to the boundary-element method,^40,41^ and it takes into account the presence of the external pulse and of the time-dependent electronic density of the molecular system.^42,43^ Even though our quantum model of the reactor is far from the experimental size, the Pd layer being 2-nm thick as shown in ref. [38], our recent study on the shape and size of Pd cluster allows us to consider the present results, specifically the sign of charge injection, as robust. Real-time calculations for electron/hole dynamics were carried out using the WaveT/TDPlas package,^17^ interfaced with AMS^44^ for extracting energies, electric transition dipole moments and electrostatic potential on the *N*_*T*_ tesserae,^38,45^ computed using TD-DFT+TB^46^ with the RPBE functional^47^ combined with Grimme corrections within a singly excited ansatz (eq 5 of ), and a double-ζ basis set. In our recent work,^38^ we have shown that B3LYP provides the same physical information, i.e., an electron injection into HCOO*, thus providing additional control over the reliability of the current results. Using more refined approaches such as GW/BSE would be computationally too demanding, since 1808 electronic states have been computed and then propagated in this work.

**Figure 1:**
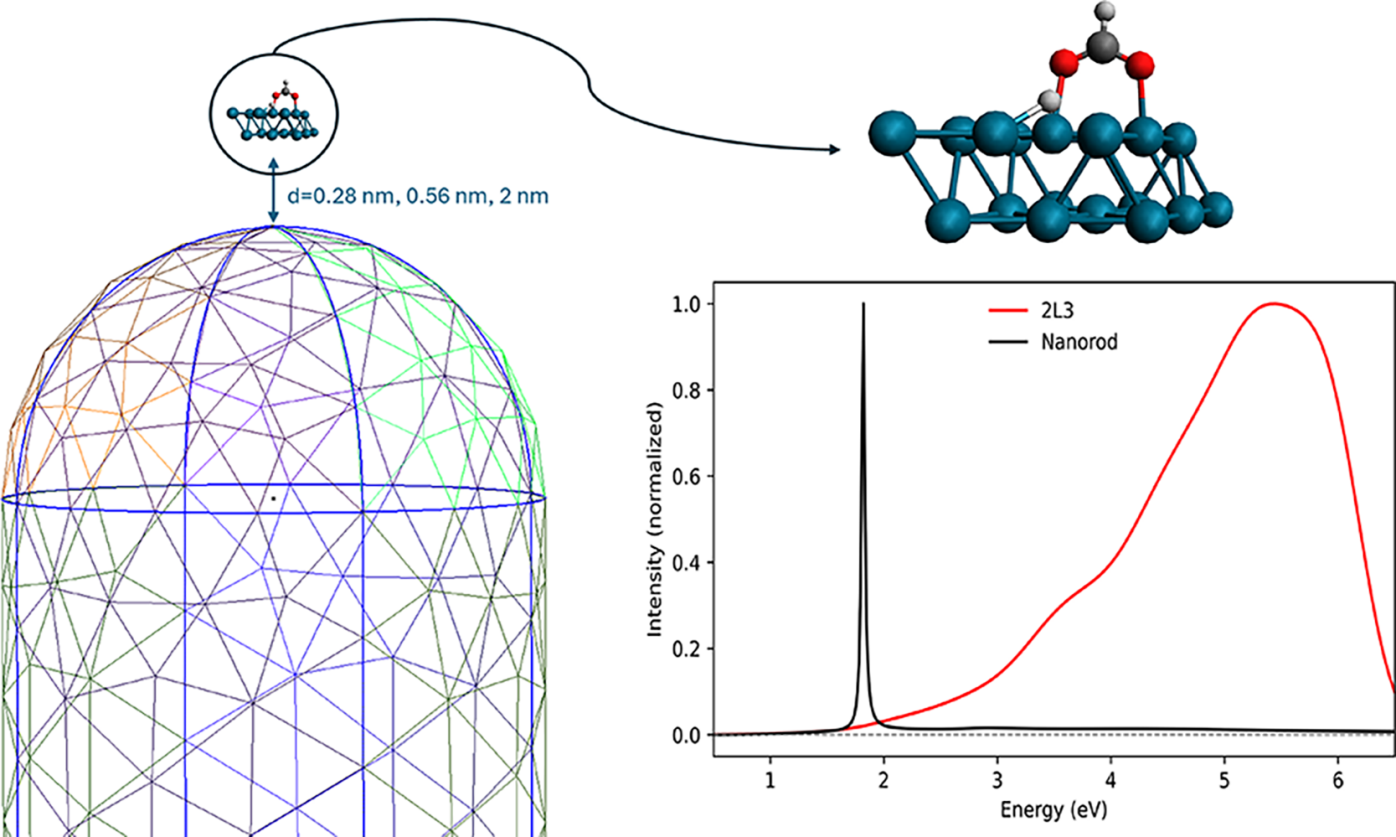
Multiscale model with a classical Au NR, and the QM subsystem. The normalized absorption spectra of the two subsystems are also reported.

In all simulations, the electric field was linearly polarized perpendicular to the Pd surface and parallel along the NR longitudinal axis. Two central frequencies for the pulse have been used: the plasmonic resonance of the NR at 1.82 eV (P1, Figure 1) and an off-resonant frequency at 3.0 eV (P2). The pulse peak intensity is equal to 10^2^ W/cm^2^, and a Gaussian envelope with a full width at half-maximum (FWHM) of 21 fs has been adopted, see eq. 6 of . This FWHM was chosen to approximate the effect of continuous-wave light with a coherence length of a few microns, consistent with experimental conditions.^10^ The photoresponse is therefore in the linear regime. Details on our theoretical approach to plasmon-mediated photocatalysis, which has been already described elsewhere,^10,29,38^ and on the computational strategy are provided in . All the simulations refer to a closed system, according to which electron dynamics is coherent. When the pulse is switched off, no other perturbation affects the electronic degrees of freedom. Effective approaches to account for charge relaxation and backtransfer could be used without an explicit nuclear dynamics.^29^ The photocatalytic pathway has been assumed to be the same occurring in the thermal reaction.^10^ Explicit nuclei were held fixed during the dynamics.

The selected distances between the Au NR and the bottom Pd layer of the QM subsystem (Figure 1) correspond to 0.28 nm, which is the sum of Au and Pd atomic radii,^48^ 0.56 and 2 nm. This last value allows us to control the decrease in plasmonic effects on the QM subsystem.

The first aspect to be addressed is the nature of the charge injection into HCOO* from the Pd layers. In our recent work on the QM subsystem only and using the P1 frequency,^38^ which is not resonant with any HCOO* excitation, we have found a net electron injection. Such charge populations (electron, hole and corresponding net one) are computed according to eqs. 8 and 9 of Supporting Information, which define differential electron and hole populations with respect to the initial, i.e. in light-off conditions, electronic density.^10,29,38^ This charge-transfer mechanism is also confirmed in the presence of the Au NR, as shown in Figure 2 for the distance of 0.28 nm. The net charge (presented as a percentage of the total charge population of the QM subsystem) on HCOO* is indeed negative. A closer inspection of the atomic contributions, given in the lower panels of Figure 2, reveals that this trend is consistent across all atoms: carbon (left), oxygen (center), and hydrogen (right). While both carbon and hydrogen atoms exhibit a modest increase in electron population, the oxygen atoms are the primary recipients of the transferred charge, indicating that they are the main targets of the electron donation process. This behavior is consistent with the previous observation in absence of a nearby plasmonic NR.^38^ However, introducing the Au NR determines a notable difference in the charge dynamics. In presence of the NR, the system experiences an additional electromagnetic contribution due to the plasmonic near field. This secondary field emerges shortly after the peak of the external pulse,^49^ with the electromagnetic response of the NR persisting for approximately 10–20 fs after the external field maximum. As a result, the QM subsystem is exposed to a prolonged and more complex electromagnetic environment. This leads to a more gradual increase in the electron and hole populations over time, producing a gentler slope in their temporal evolution compared to the case without the NR (for comparison, see Figure 4 of ref. 38). Additional evidence supporting this interpretation is given by the results of the control simulation presented in , where the P2 pulse (central frequency of 3 eV) has been used. Despite the NR being present, the electron and hole populations exhibit the same sharp rise and rapid plateau observed in the absence of NR. This comparison further confirms that the prolonged and nontrivial charge redistribution observed here is specifically driven by resonant coupling between the external field and the NR plasmonic excitation.

**Figure 2:**
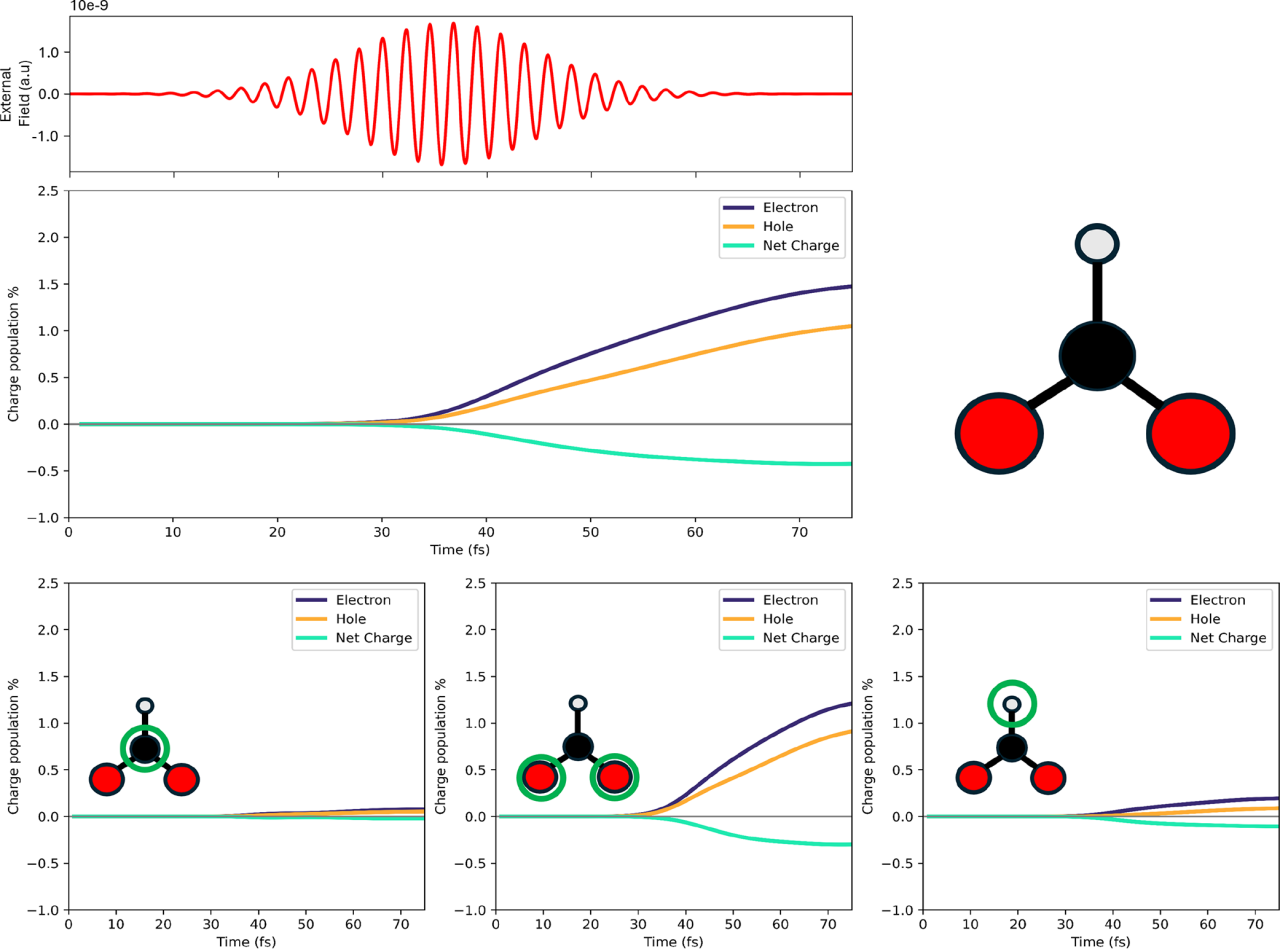
Upper panels: external P1 pulse and time evolution of the photoinduced charge populations (electron, hole and net) of the HCOO* fragment in the presence of the Au NR at 0.28 nm. Bottom panels: on the left, time evolution of the photoinduced charge populations (electron, hole and net) of the carbon atom; in the middle, the same for the oxygen atoms; on the right, the same for the hydrogen atom of HCOO*.

Moreover, presents the evolution of the electron and hole populations within the Pd cluster over the first 75 fs of real-time dynamics with the P1 pulse: an overall positive net charge is observed, as expected, since no source of sink of charges is present in the simulation.

By varying the frequency of the external pulse, we are able to disentangle in the electronic dynamics of the QM subsystem the contributions of direct polarization effects from those arising due to the plasmonic excitation. In Figure 3, we report the time evolution of the net charge on both the Pd cluster and on HCOO*+H* for three configurations: (i) with the NR present and excited about the plasmonic resonance (P1 pulse), (ii) with the NR excited off-resonance (P2 pulse), and (iii) in the absence of the NR with the P1 pulse (data taken from ref 38). Results for the two first cases refer to the distance of 0.28 nm.

**Figure 3:**
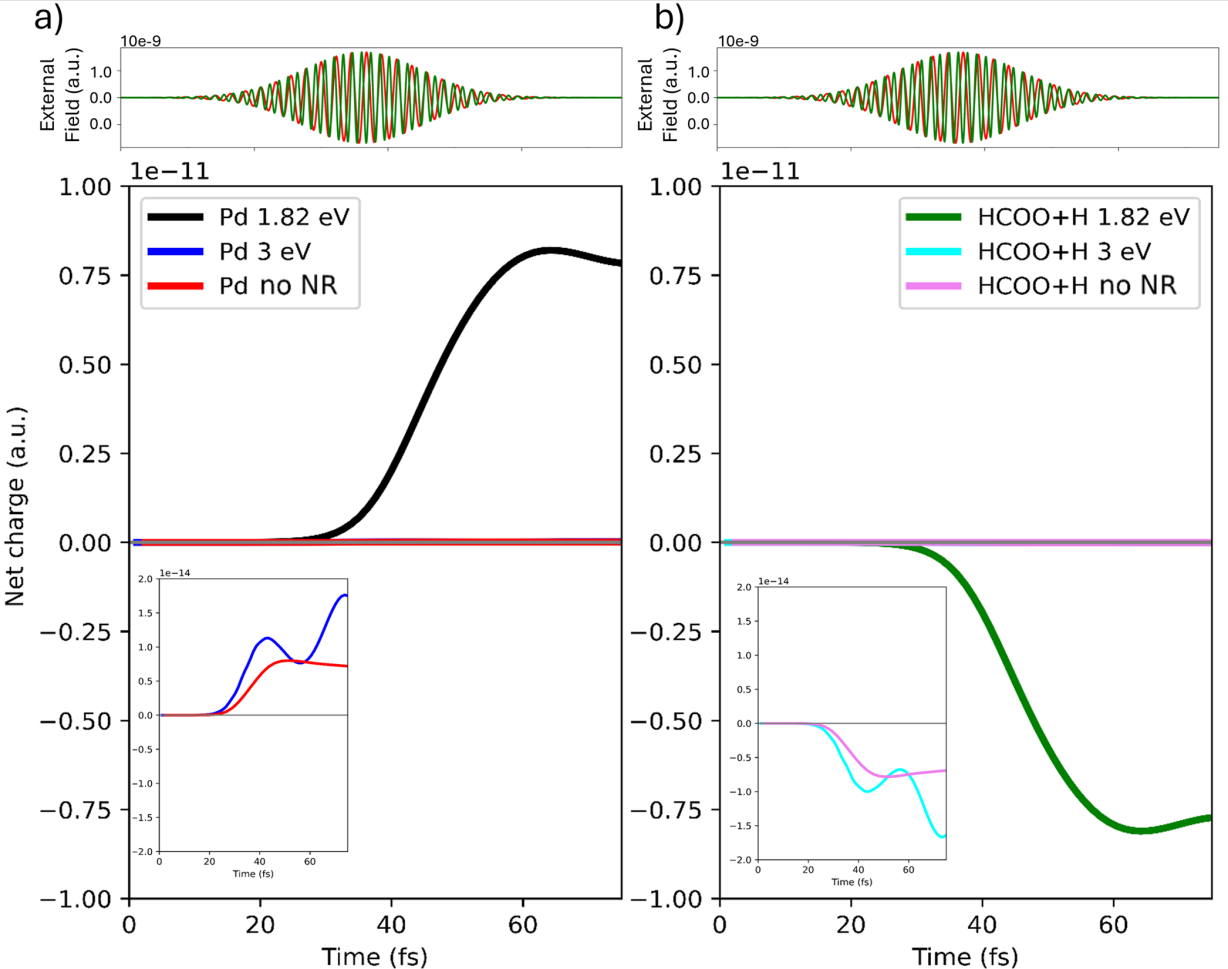
Panel a): time evolution of the net charge of the Pd cluster at 0.28 nm from the NR with the P1 pulse (reported in red), with the P2 pulse (reported in green) and without NR. Panel b): time evolution of the net charge of the HCOO* and H* species at 0.28 nm from the NR with P1 pulse, with P2 pulse and without NR. In both cases, a zoom for the second and third configurations is also shown. P1 (red) and P2 (green) pulses are also shown.

The interaction at the plasmonic resonance (1.82 eV) produces a net charge transfer that is approximately 3 orders of magnitude larger than that observed in the off-resonant case or when the NR is not present. Also, the temporal profile of the curves is different, indicating an active role played by the plasmonic field during the dynamics. Indeed, the differential projected DOS (ΔPDOS, eq. 7 of Supporting Information) of HCOO* collected in and for P1 and P2 pulses at 0.28 nm and for *t* = 60 fs (tail of the pulse), respectively, shows a different depopulation/population of molecular orbitals: with the P2 pulse, lower-energy orbitals are depopulated and virtual ones at higher energy are populated, as expected. In both cases, the involved orbitals exhibit a hybrid character, with electron density delocalized between the Pd atoms and the molecular fragment. A snapshot at 60 fs has been taken.

We have also analyzed how varying the distance between the QM subsystem and the NR influences the observed electron (hole) injection into HCOO* (Pd atoms). This analysis is presented in , which shows the total charge displacement induced by the P1 pulse for the three configurations with increasing separation: 0.28, 0.56, and 2 nm. The total charge displacement at 0.28 nm is nearly 1 order of magnitude larger than that at 2 nm. Despite these quantitative differences, the overall charge transfer mechanism remains qualitatively consistent across all distances. As shown in , the spatial distribution of electron and hole populations for both the Pd layers and the adsorbed HCOO* follows the same pattern at 0.28, 0.56, and 2 nm. This suggests that the primary effect of decreasing the distance is not to alter the nature of the electron transfer process, but rather to amplify it via stronger electromagnetic coupling with the NR. Comparing the ΔPDOS of HCOO* at three distances (0.28, 0.56, and 2 nm, ) under excitation by the P1 pulse, one observes that the most striking difference lies in the intensity of the peaks, which are all scaled homogeneously.

The analysis conducted so far does not yet provide elements to understand the increase in H_2_ production due to plasmonic effects. As mentioned above, the bidentate adsorbed HCOO* species becomes monodentate, with an oxygen atom released from the Pd surface. Figure 4 shows the asymmetry of electron injection into the two oxygen atoms of HCOO*, in terms of the absolute value of the relative difference.

**Figure 4:**
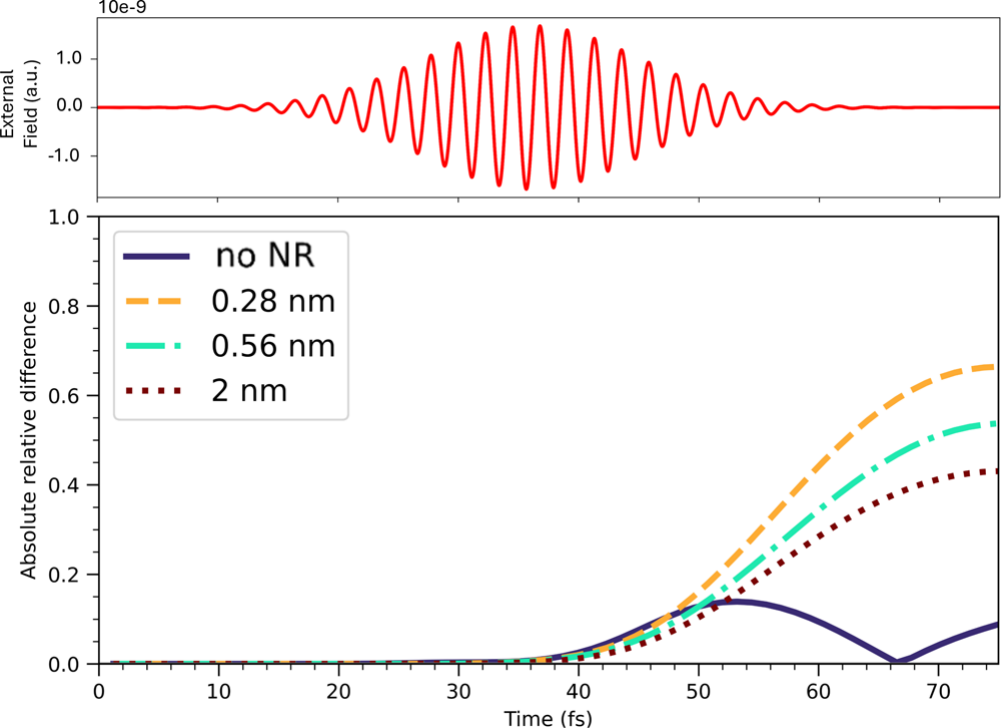
Absolute relative difference of the electron population of the two O atoms of HCOO* with the P1 pulse, at various distances between the QM subsystem and the NR. The case of the isolated QM subsystem is also reported for comparison. The time profile of the pulse is given.

Two different behaviors can be inferred. The asymmetry becomes more pronounced as a function of time by decreasing the distance, being maximum for the configuration at 0.28 nm, at larger times, when the plasmonic field becomes active.

It is the inner oxygen atom to be less negatively charged at any time in the presence of the NR, and therefore the candidate to leave the Pd surface (Figure S10). The two oxygen atoms are labeled as shown in . Instead, without the NR, the time profile is rather different and is characterized by a change in the asymmetry (the singularity at around 67 fs). Distances are 2.12 (2.18) Å for the O1 (O2) O atom. Asymmetry is inherent in the system, as can be seen from the result without NR: an asymmetrical charge population is present due to the adsorption of HCOO* on the Pd surface. We emphasize that, as described in detail in the Supporting Information, the geometry optimization was conducted in periodic boundary conditions, starting from literature data. The presence of NR increases the asymmetry, thus leading more quickly to the monodentate species and to the final product, i.e., H_2_. In , we present the same analysis using the P2 pulse. In the absence of the NR, a slight asymmetry emerges, similar to what is observed with the P1 pulse without NR. This behavior is expected, as the system naturally tends toward the monodentate configuration even under excitation with the P2 pulse. Importantly, the presence of the NR does not alter this asymmetry: the charge distribution remains essentially unchanged when reducing the catalyst-NR distance from 2 to 0.28 nm with the P2 pulse, closely resembling the case without NR. This observation is highly significant, as it highlights the central role of plasmonic excitation in the H_2_ enhancement observed in this reaction.

To verify that the asymmetry described is not an artifact due to the choice of the QM subsystem, we calculated the charge population on the two oxygen atoms for the isolated cluster (i.e., no NR) with two layers of Pd, with 16 atoms per layer (2L4), reported in . Asymmetry in 2L4 is much larger than in 2L3. The reason for that is the two atoms being characterized by a net charge of opposite sign, with the electron one larger in absolute value (). This result depends on the shape of the molecular orbitals involved in the dynamics, which differ from those in the 2L3 case, where both oxygen atoms are negatively charged and border effects play a non-neglible role. In 2L4, finite-size effects should be partially reduced, since the two oxygen atoms are centered with respect to the cut surface.

Assuming that the asymmetry in the photoinduced charge distribution is the driving force for hydrogen formation, our results confirm that this effect becomes even more pronounced under plasmonic conditions—specifically at 0.28 nm and with the excitation resonant with the NR LSPR. This provides a microscopic explanation for the enhanced H_2_ production rate.

We have also analyzed the charge evolution of the H* atom. Figure S14 of shows the time-resolved electron and hole populations for the hydrogen fragment under P1 pulse at a separation of 0.28 nm: a net electron injection is observed, as in the case without NR.^38^

Surface-charge heterogeneity of the Pd surface has been identified as a key factor contributing to photocatalytic enhancement, as it can promote substrate adsorption and stabilize reaction intermediates.^50^ According to Zheng et al.,^13^ this heterogeneity plays a central role in explaining the increased catalytic activity observed in the presence of a plasmonic nanostructure.

Panel a) of Figure 5 shows the net charge distribution over selected pairs of Pd atoms located in the upper layer of the slab when the system is separated by 0.28 nm from the nanorod and excited with the P1 pulse. The pair marked in blue becomes more positively charged, while the purple pair accumulates negative charge. The central pair (green curve), which directly interacts with HCOO*, remains nearly neutral. Panel b) of Figure 5 explores how this surface-charge pattern changes with distance from the NR. Although the qualitative shape of the charge distribution remains similar, the total amount of displaced charge is significantly decreased—by about 1 order of magnitude—when the distance is increased to 2 nm, again under P1 excitation. Panels (c) and (d) of Figure 5 extend this comparison to the nonresonant case (P2 pulse), where the system is no longer excited at the plasmonic frequency. In these conditions, the charge separation on the Pd surface is much smaller (2 orders of magnitude) than what is observed in resonant conditions at the corresponding distances. On the other hand, the spatial pattern remains consistent when changing the distance. The data in Figure 5 are reported as both percentage and absolute values.

**Figure 5:**
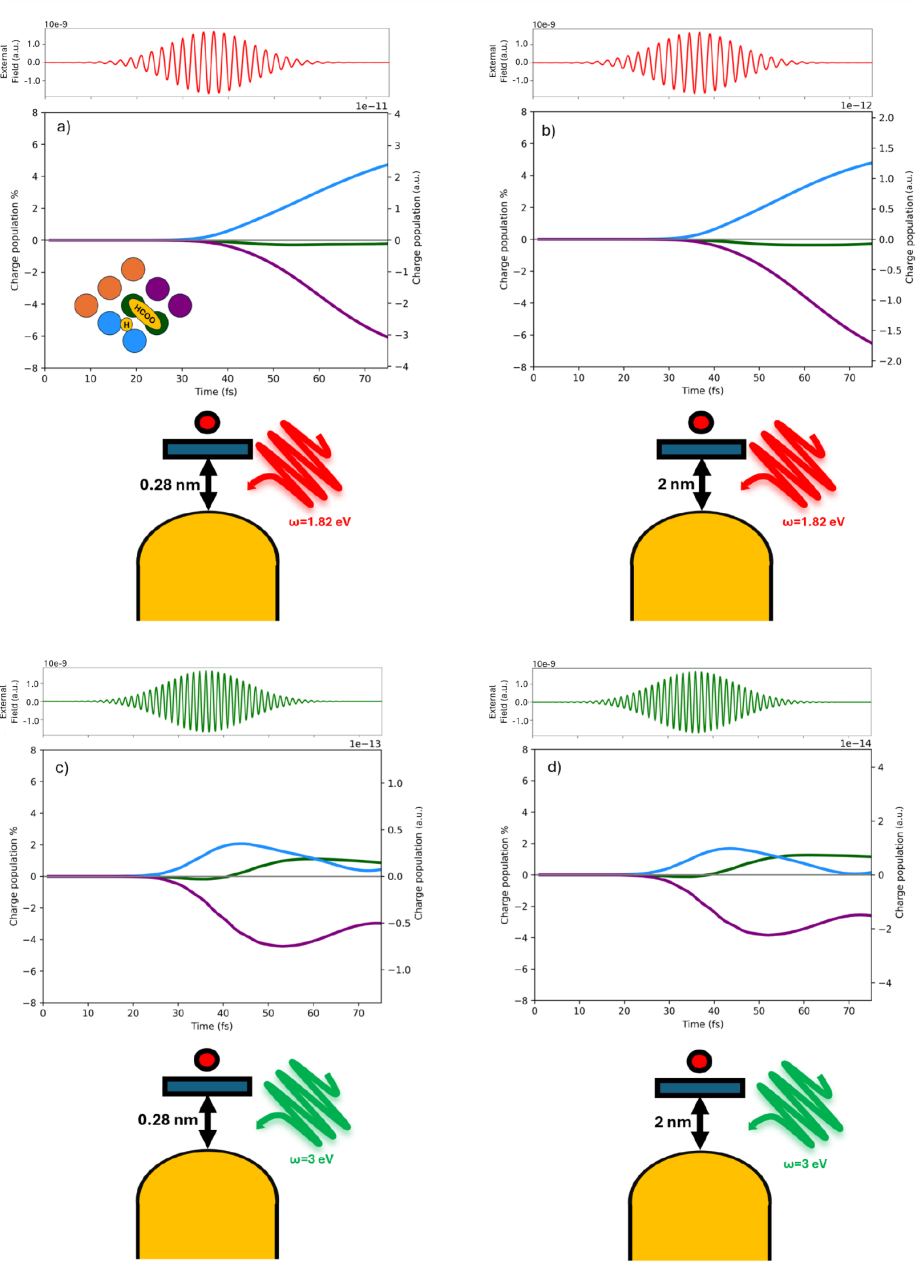
Panel a) Time evolution of the photoinduced charge populations (electron, hole and net) of the Pd atoms of the upper layer interacting with the molecular species at 0.28 nm with P1 pulse reported on top of each graph. In green are the Pd pairs interacting with HCOO*, in blue Pd pairs interacting with H* and in purple Pd pairs not interacting with any molecular species. Panel b) at 2 nm from NR with P1 pulse. Panel c) at 0.28 nm from the NR with P2 pulse. Panel d) at 2.0 nm from the NR with P2 pulse.

In this work, we have provided an original interpretation of the plasmon-mediated enhancement of hydrogen production from formic acid in the presence of a Pd-tipped Au NR. Our analysis shows that (i) a net electron charge is injected from Pd atoms to HCOO*, which is the reaction intermediate; (ii) the asymmetry in the charge distribution on the oxygen atoms is maximum in plasmon-resonance conditions, thus explaining how the plasmonic field affects the hydrogen production rate; (iii) a greater spatial heterogeneity of the charge on the Pd surface was found in the case of resonance with the plasmon of the NR. Since the plasmonic field decays in 10−20 fs, we have assumed that nuclear motion is decoupled from the electron dynamics occurring in this time window, assuming the reaction pathway from literature. Though freezing nuclei can be a crude assumption, however, we believe that reliable information about the plasmonic role in modifying reaction steps can be provided by such a modeling.

## Supplementary Material





## Data Availability

Data are available from the authors (L.B. and E.C.) upon reasonable request.

## Notes

Theoretical approach and computational details. Charge population of HCOO* at 0.28, 0.56, and 2 nm and with P2 pulse (results for 0.56 and 2 nm also with P1). Charge population of Pd atoms at 0.28, 0.56, and 2 nm and with P1 and P2 pulse. ΔPDOS of HCOO* at various configurations. Net total charge at various configurations. Charge population of O atoms at 0.28 nm and with P1 pulse. Absolute relative difference of charge population of O atoms in different conditions, also for the 2L4 subsystem. Charge population of H* at 0.28 nm and with the P1 pulse (PDF)
Transparent Peer Review report available (PDF)
