# Supplementary material for: Plasmon-Enhanced Asymmetry in the Charge Distribution Explains the Increased H2 Production Rate from Formic Acid with a Pd-Tipped Au Nanorod
Source: J Phys Chem Lett. 2025 Dec 11;16(50):12931–8. doi: 10.1021/acs.jpclett.5c02858 (PMC12720282; doi:10.1021/acs.jpclett.5c02858)
Supplement: Supplementary file 1 [file jz5c02858_si_001.pdf]

Supporting Information for "Plasmon-enhanced asymmetry in the charge distribution explains the increased H_2 production rate from formic acid with a Pd-tipped Au nanorod"

Leonardo Biancorosso and Emanuele Coccia*

Dipartimento di Scienze Chimiche e Farmaceutiche, Università di Trieste, via L. Giorgieri 1, 34127, Trieste, Italy

E-mail: ecoccia@units.it

Theory and computational details

TDSE in length gauge for TD-PCM-NP can be rewritten as

$$i\frac{d}{dt}|\Psi(t)\rangle = \hat{H}(t)|\Psi(t)\rangle, \tag{1}$$

with $\hat{H}(t)$ being the time-dependent Hamiltonian

$$\hat{H}(t) = \hat{H}_0 - \hat{\vec{\boldsymbol{\mu}}} \cdot \vec{E}_{ext}(t) + \mathbf{q}(t) \cdot \hat{\mathbf{V}}_{\mathrm{BEM}}. \tag{2}$$

In Eq. 2, $\hat{\vec{\boldsymbol{\mu}}}$ denotes the dipole operator of the molecule, $\vec{E}_{ext}(t)$ represents the time-dependent external electric field, $\mathbf{q}(t)$ corresponds to the time-dependent BEM charges,[1,2] and $\hat{\mathbf{V}}_{\mathrm{BEM}}$ is the operator describing the electrostatic potential evaluated at the positions of the sur-

face charges on the nanoparticle (NP). Both $\mathbf{q}(t)$ and $\hat{\mathbf{V}}_{\text{BEM}}$ are vectors of dimension N_T, corresponding to the number of surface elements.

The field-free electronic Hamiltonian, $\hat{H}_0$, is given by:

$$\hat{H}_0 = \hat{H}_{el} + \mathbf{q}_{GS} \cdot \hat{\mathbf{V}}_{\text{BEM}} \tag{3}$$

where $\hat{H}el$ is the electronic Hamiltonian of the isolated QM subsystem, and $\mathbf{q}_{GS}$ denotes the ground-state BEM charges. These charges are determined through a self-consistent procedure that accounts for the polarization response of the nanoparticle to the molecular ground-state electronic density.[3]

The time-dependent wavefunction, $|\Psi(t)\rangle$, is defined as a linear combination of the N_{states} eigenstates of the effective field-free Hamiltonian $\hat{H}_0$, also containing the ground-state polarization of the molecule and the NP

$$|\Psi(t)\rangle = \sum_{M=0}^{N_{\text{states}}-1} C_M(t)|M\rangle \tag{4}$$

where $C_M(t)$ are time-dependent coefficients, and $|M\rangle$ is the M-th eigenstate of the system, with eigenvalue E_M. The initial guess for the self-consistent calculation is provided by the eigenstates of H_{el}.

In this work, we employ an approximate time-dependent density functional theory approach with tight-binding corrections (TD-DFT+TB),[4] formulated within a configuration-interaction singles (CIS) framework,[5] as implemented in the Amsterdam Modeling Suite (AMS).[6] Within this formalism, the excited states $|M\rangle$ are expressed as linear combinations of singly excited Slater determinants:

$$|M\rangle = \sum_{i}^{occ}\sum_{a}^{vir} d_{i,M}^{a}|\Phi_i^a\rangle \tag{5}$$

where $|\Phi_i^a\rangle$ represents a determinant formed by promoting one electron from an occu-

pied molecular orbital i to a virtual orbital a, and $d_{i,M}^{a}$ are the corresponding excitation amplitudes.

The system is perturbed by a time-dependent external electric field $\vec{E}_{ext}(t)$, defined as:

$$\vec{E}_{ext}(t) = \vec{E}_{max} \exp\left(-\frac{(t-t_0)^2}{2\sigma^2}\right) \sin(\omega t), \tag{6}$$

where $\vec{E}_{max}$ denotes the peak field amplitude, ω is the carrier frequency, t_0 is the temporal center of the pulse, and σ controls the width of the Gaussian envelope.

In order to analyze the electronic dynamics, we use the time-dependent projected density of states (PDOS(t,ϵ)),[7] defined as the expectation values of the number operator $\hat{n}$ with respect to the wavefunction $|\psi(t)\rangle$. In particular, we define the differential PDOS (ΔPDOS) at a certain time t in respect to the initial condition at time $t = 0$.

$$\begin{aligned}\Delta\text{PDOS}_K(t,\epsilon) = &-\sum_{i}^{occ} w_i^K \text{Re}[\sum_{M,L} C_L^*(t) C_M(t) \sum_{a}^{vir} d_{i,L}^{a*} d_{i,M}^{a}] F_\eta(\epsilon-\epsilon_i) \\ &+\sum_{a}^{vir} w_a^K \text{Re}[\sum_{M,L} C_L^*(t) C_M(t) \sum_{i}^{occ} d_{i,L}^{a*} d_{i,M}^{a}] F_\eta(\epsilon-\epsilon_i).\end{aligned} \tag{7}$$

In this equation, $d_{i,M}^{a}$ ($d_{i,L}^{a}$) are the linear coefficients of the expansion for state $|M\rangle$ ($\langle L|$) and F_η is a Lorentzian function centered on the MO energies ϵ_i, with width η, used to obtain a smooth profile. Mulliken weights w_i^K are used in the fragmentation of the studied system. Details are found in Ref. 7. The subscript/superscript K refers to the fragment, i.e. the single atom or group of atoms, regarding which one computes the charge population.

Integrating over the energy range allows us to obtain the time-dependent charge (electron and hole) population regarding the initial condition[8,9]

$$\text{electron population} = \frac{1}{2}\int_{-\infty}^{+\infty} [\Delta\text{PDOS}_K(t,\epsilon) + |\Delta\text{PDOS}_K(t,\epsilon)|] d\epsilon \tag{8}$$

and

$$\text{hole population} = \frac{1}{2}\int_{-\infty}^{+\infty} \left[\Delta\text{PDOS}_K(t,\epsilon) + |\Delta\text{PDOS}_K(t,\epsilon)|\right] d\epsilon. \tag{9}$$

The geometries of the Pd(111) slab and the molecular species were taken from the optimized structures reported in Ref.[10] Two layers and 9 atoms per layer with HCOO* and H* form the molecular system (2L3). Real-time electronic dynamics were simulated using the `WaveT/TDPlas` package,[11] interfaced with `AMS` to extract both electric transition dipole moments and the transition potential.[7,12,13] Input parameters—including excitation energies, transition dipole moments, and transition potentials—were obtained from TD-DFT+TB calculations[4] including 1808 excited states covering excitations up to 6 eV, using the RPBE exchange-correlation functional,[14] Grimme dispersion corrections, and a double-zeta (DZ) basis set.

Simulations were carried out for a duration of 100 fs. The external field $\vec{E}_{\text{ext}}(t)$ is described by Eq. 6. The plasmonic frequency was computed using the boundary element method (BEM) coupled with a polarizable continuum model.[11]

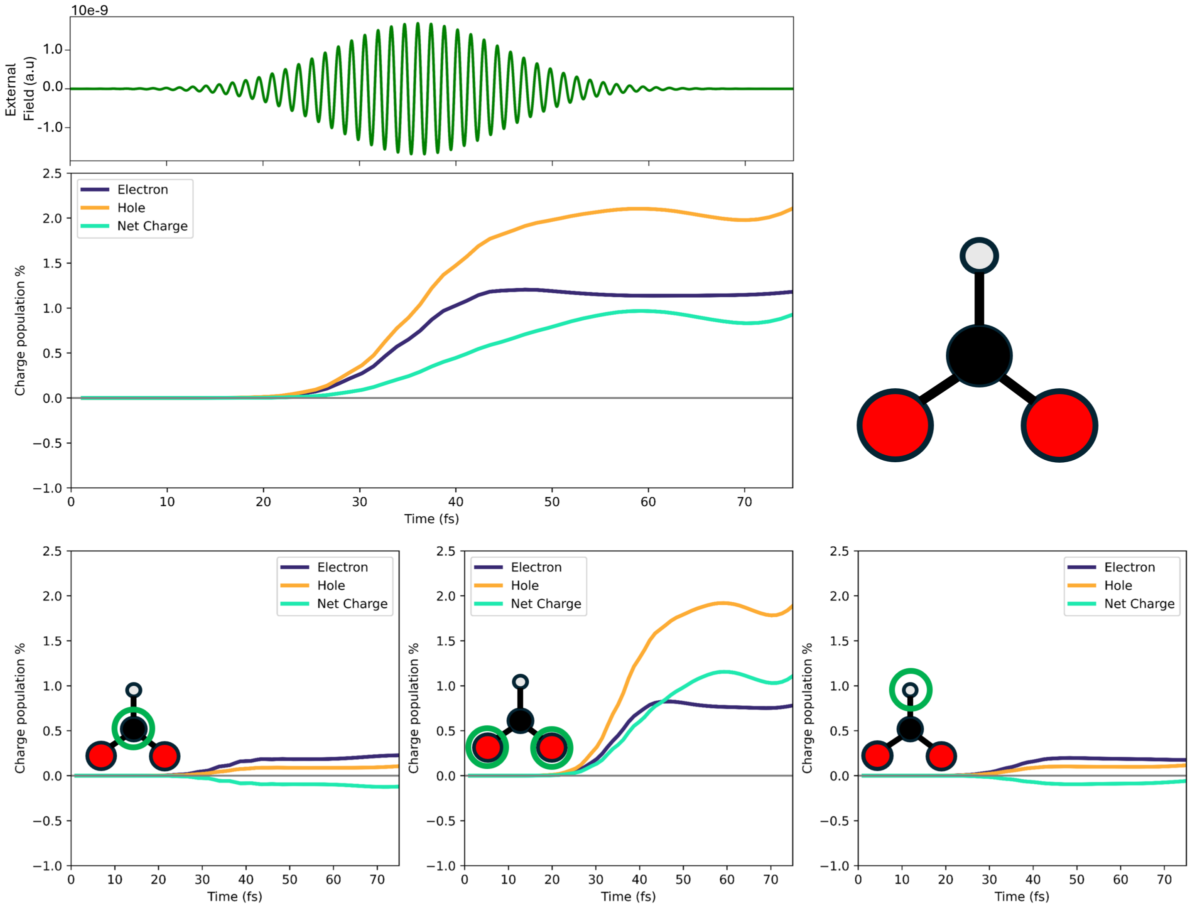

Figure S1: Upper panels: external pulse and time evolution of the photoinduced charge populations (electron, hole and net) of the HCOO* fragment in the presence of the Au NR at 0.28 nm of distance with P2 pulse reported on top. Bottom panels: on the left, time evolution of the photoinduced charge populations (electron, hole and net) of the carbon atom; in the middle, the same for the oxygen atoms; on the right, the same for the hydrogen atom of HCOO*.

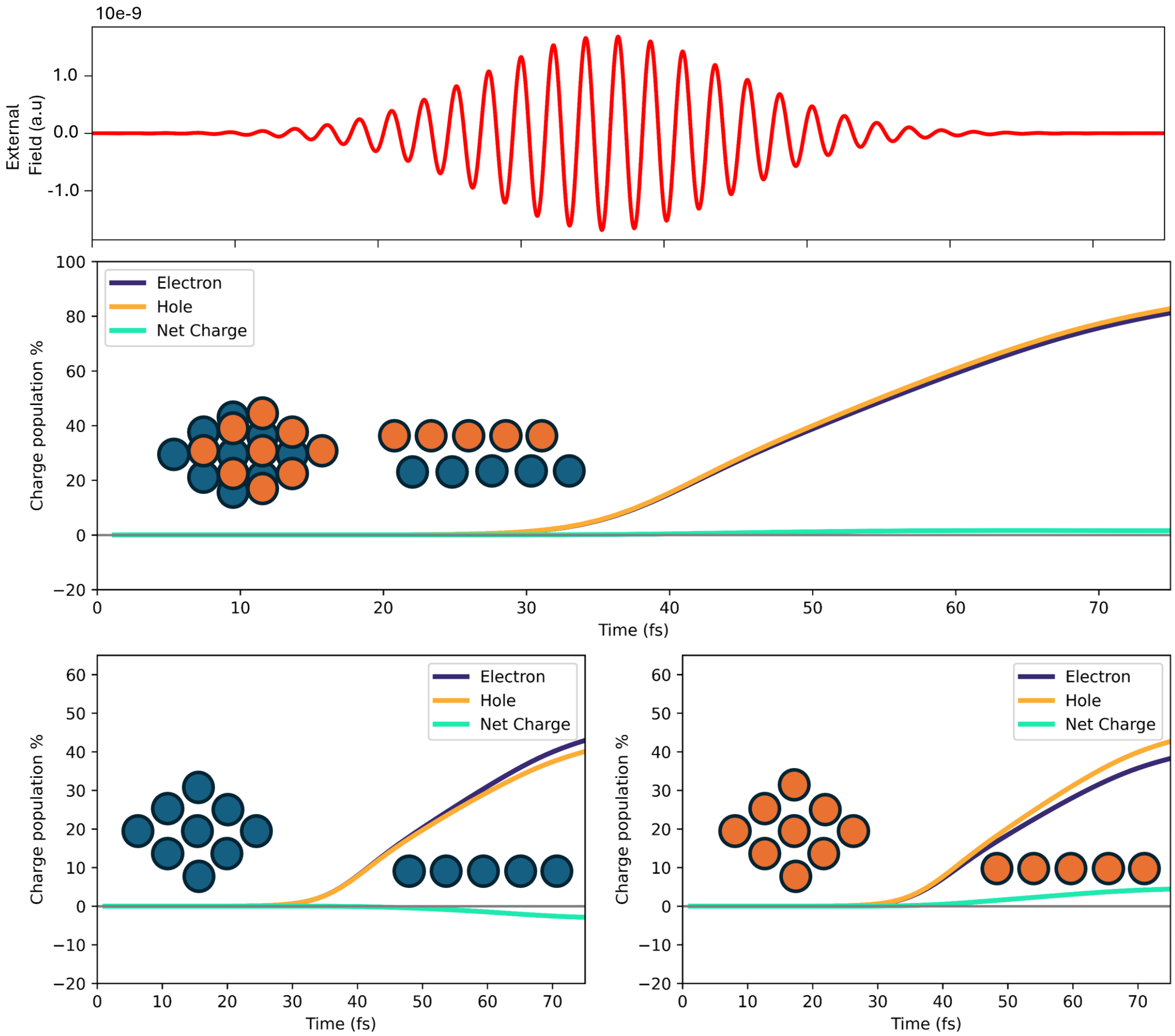

Figure S2: Upper panels: external pulse and time evolution of the photoinduced charge populations (electron, hole and net) of the full Pd cluster in the 2L3 system in the presence of the Au NR at 0.28 nm of distance with P1 pulse. Bottom panels: time evolution of the photoinduced charge populations (electron, hole and net) of the bottom layer (left) and of the upper layer (right)

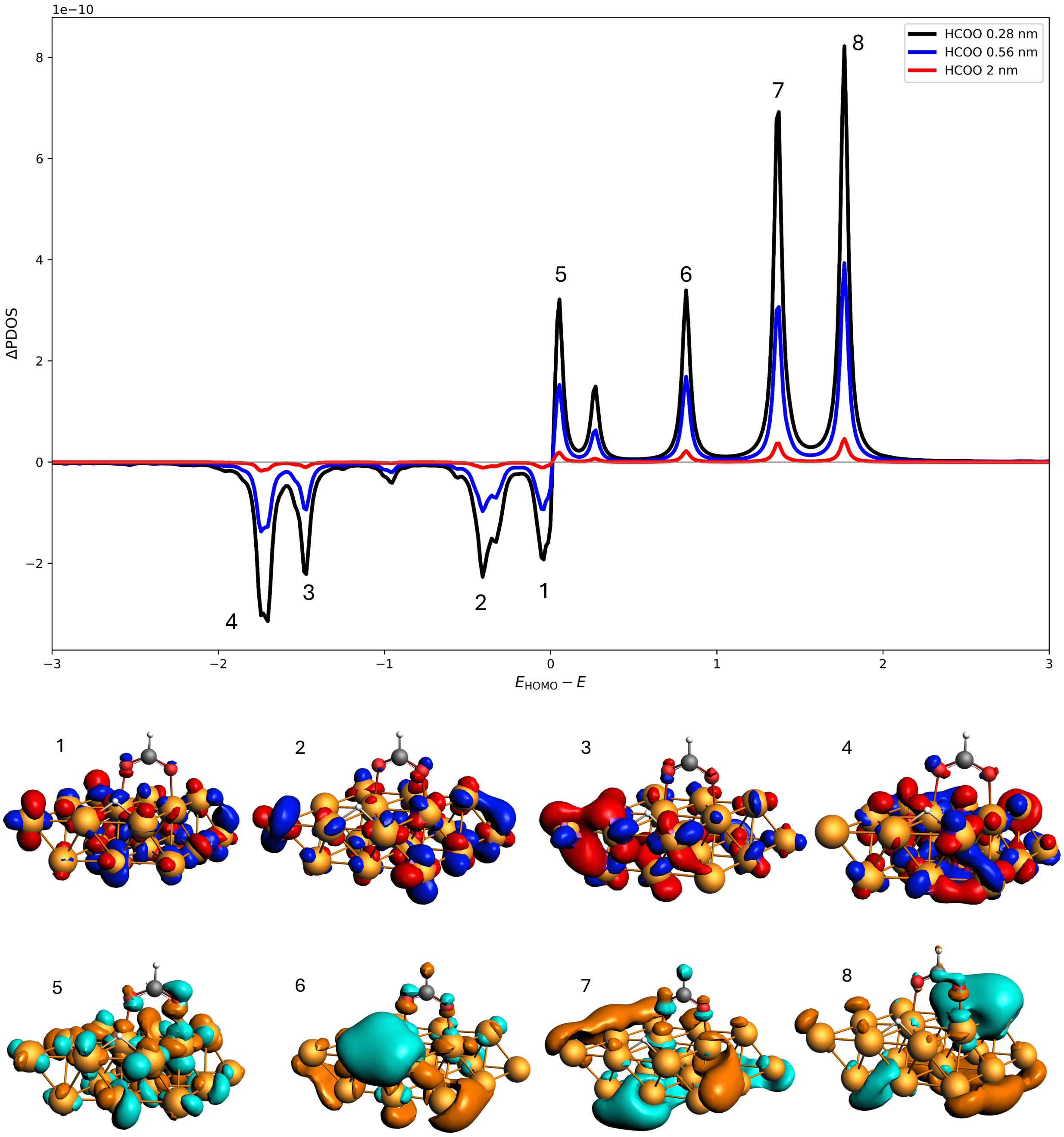

Figure S3: ΔPDOS of the HCOO* fragment at $t = 60$ fs for the system at 0.28 nm, 0.56 nm and 2 nm with the P1 pulse. In the inset, ΔPDOS of the HCOO* fragment at $t = 60$ fs for the system without NR. Reported are the main orbitals involved in the electronic process.

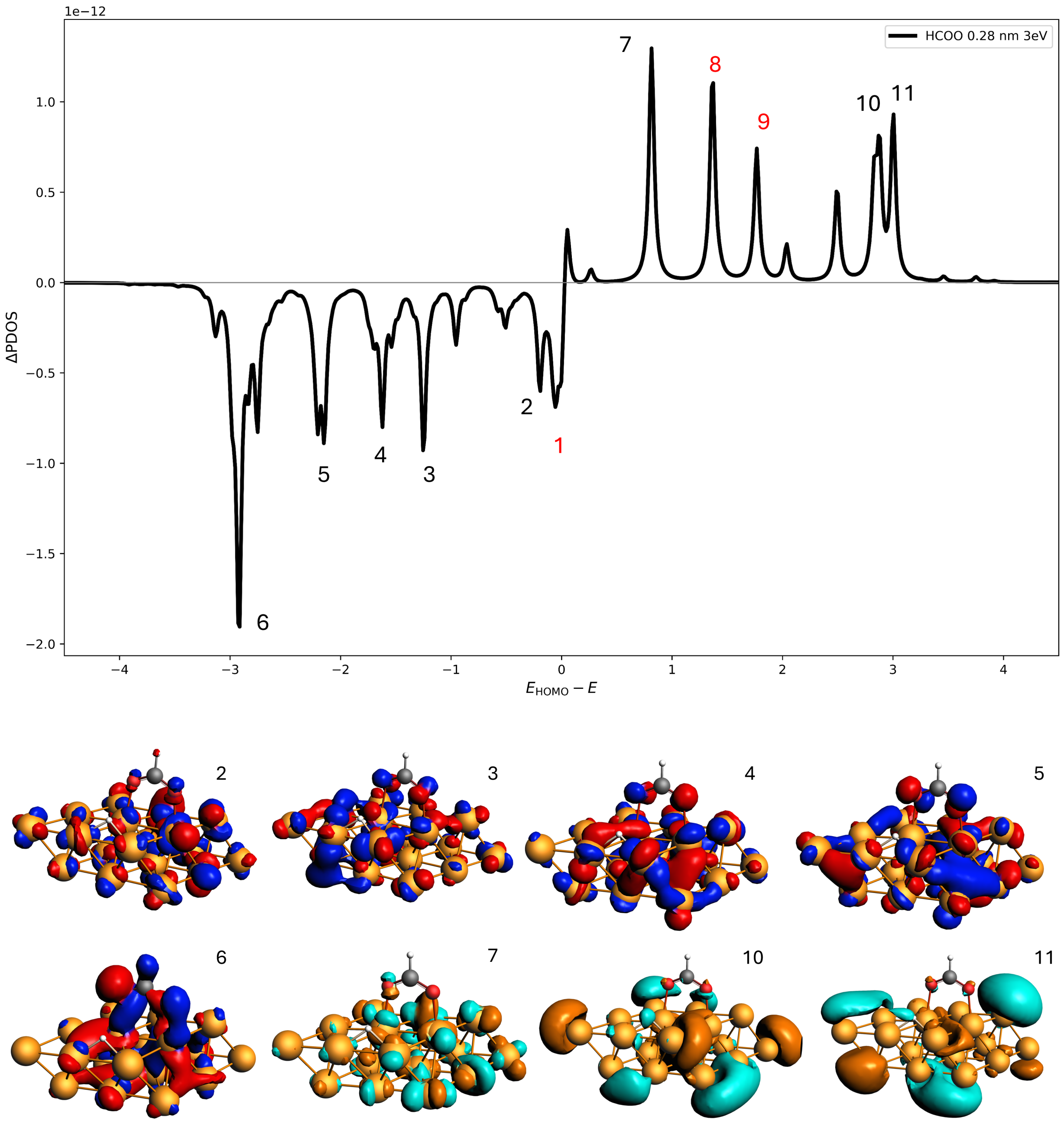

Figure S4: ΔPDOS of the HCOO* fragment at $t = 60$ fs for the system at 0.28 nm with the P2 pulse. Reported are the main orbitals involved in the electronic process. In red orbital 1, 8 and 9 are the same orbitals already reported in Figure 6 and correspond, respectively, to orbitals 1, 6 and 7.

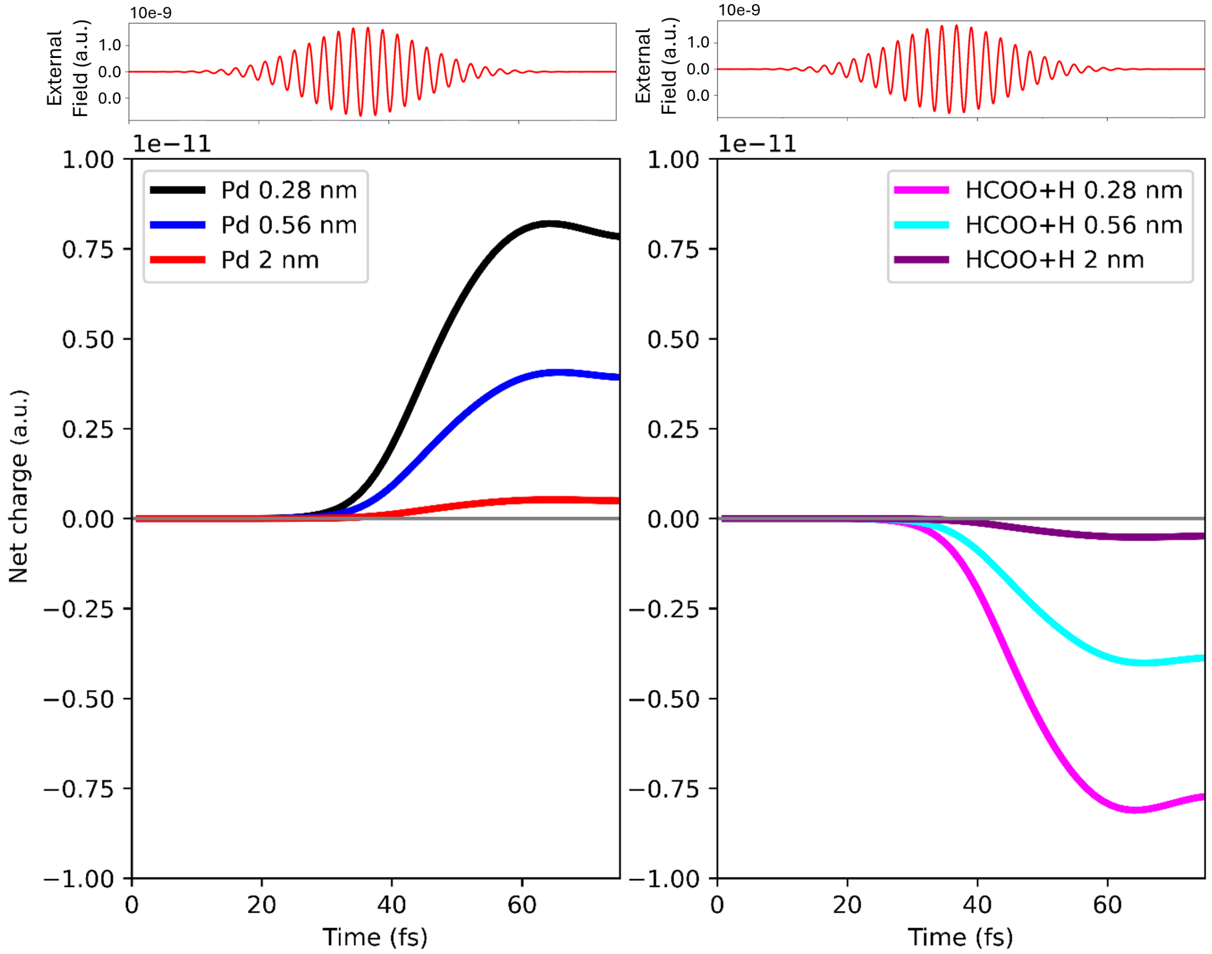

Figure S5: On the left, time evolution of the net charge of the Pd cluster at 0.28 nm, 0.56 nm and 2 nm from the NR with the P1 pulse; on the right, time evolution of the net charge of the HCOO* fragment at 0.28 nm, 0.56 nm and 2 nm from the NR with the P1 pulse. Time profile of the pulse is also reported.

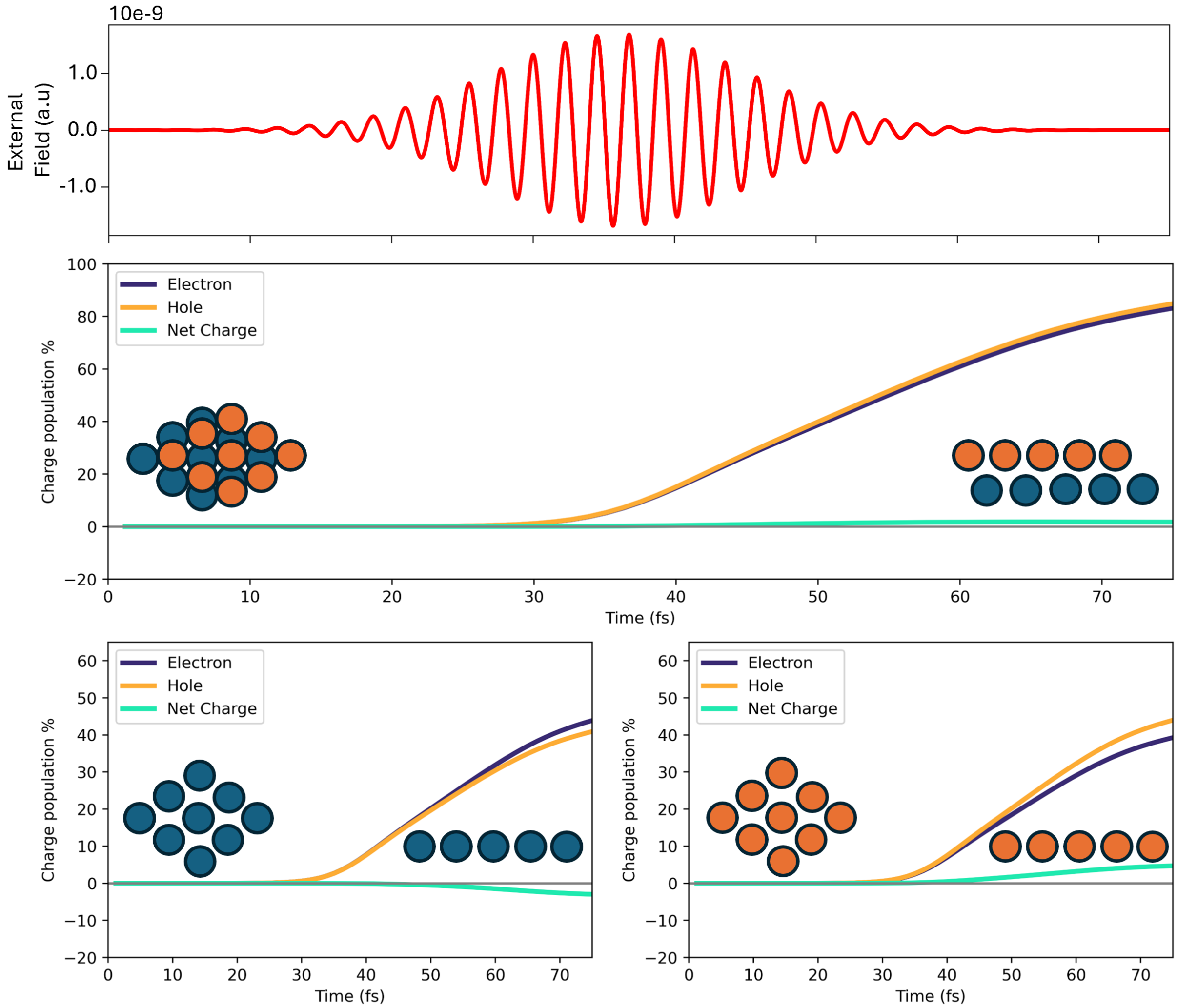

Figure S6: Upper panels: external pulse and time evolution of the photoinduced charge populations (electron, hole and net) of the full Pd cluster in the presence of the NR at 0.56 nm with P1 pulse. Bottom panels: time evolution of the photoinduced charge populations (electron, hole and net) of the bottom layer (left) and of the upper layer (right)

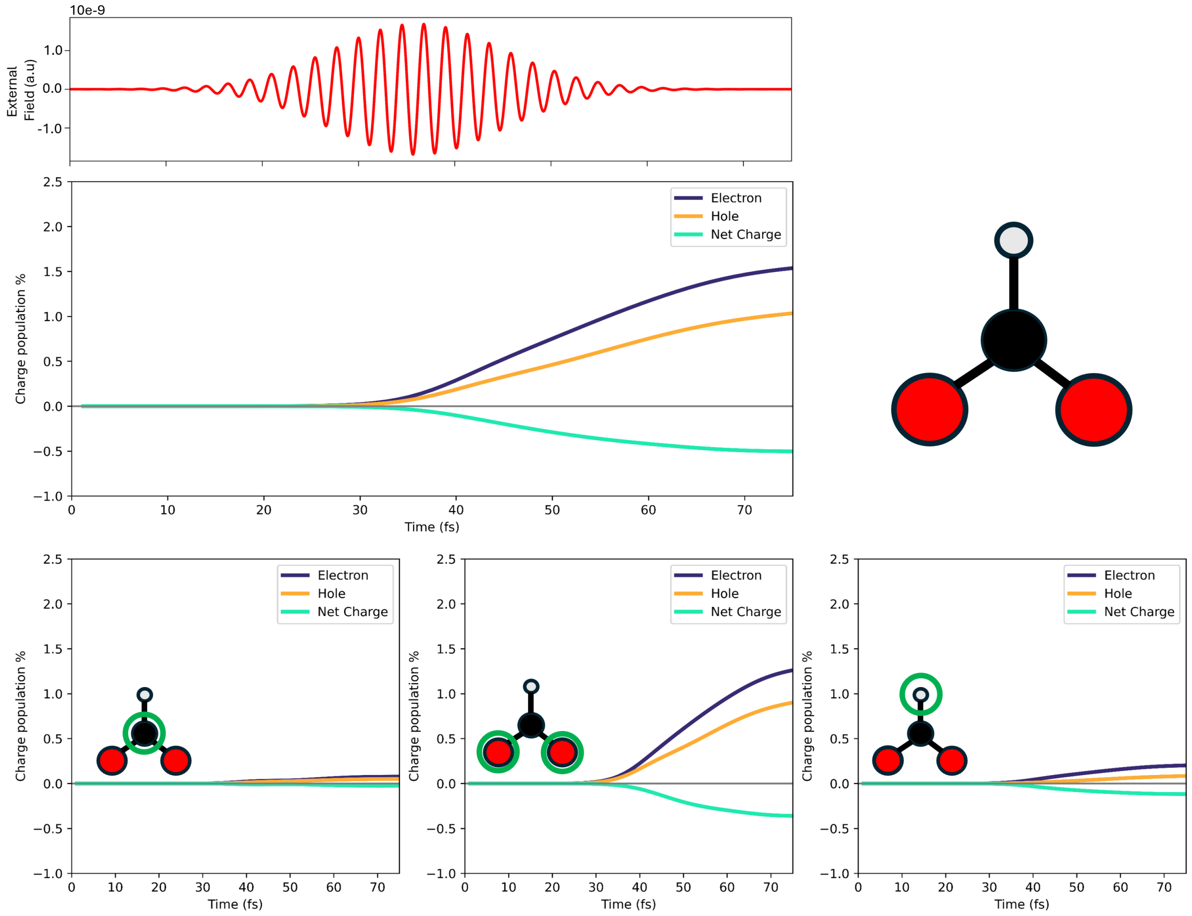

Figure S7: Upper panels: external pulse and time evolution of the photoinduced charge populations (electron, hole and net) of the HCOO* fragment in presence of the NR at 0.56 nm with P1 pulse. Bottom panels: on the left, time evolution of the photoinduced charge populations (electron, hole and net) of the carbon atom; in the middle, the same for the oxygen atoms; on the right, the same for the hydrogen atom of HCOO*.

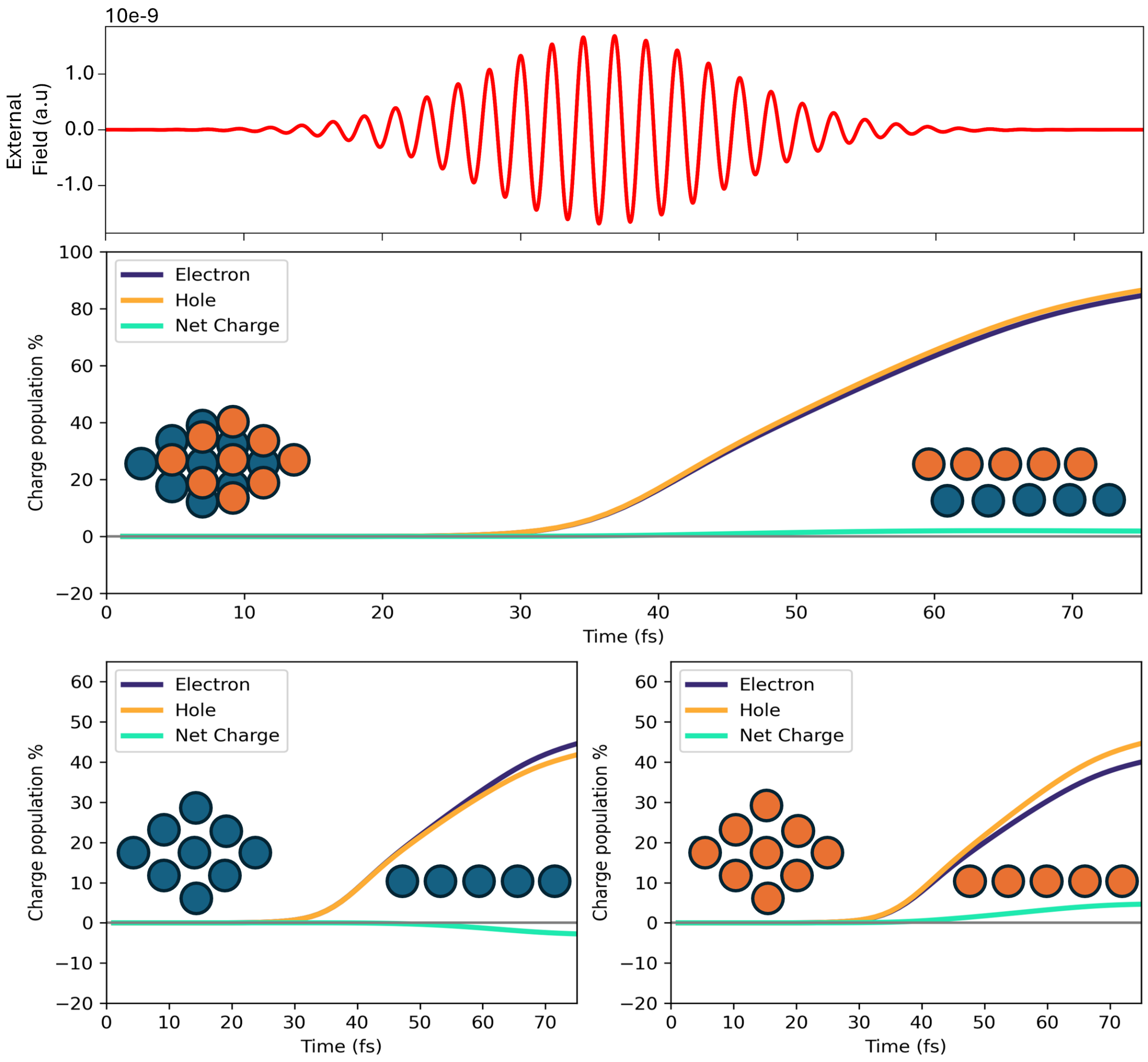

Figure S8: Upper panels: external pulse and time evolution of the photoinduced charge populations (electron, hole and net) of the full Pd cluster in presence of the NR at 2 nm with P1 pulse. Bottom panels: time evolution of the photoinduced charge populations (electron, hole and net) of the bottom layer (left) and of the upper layer (right)

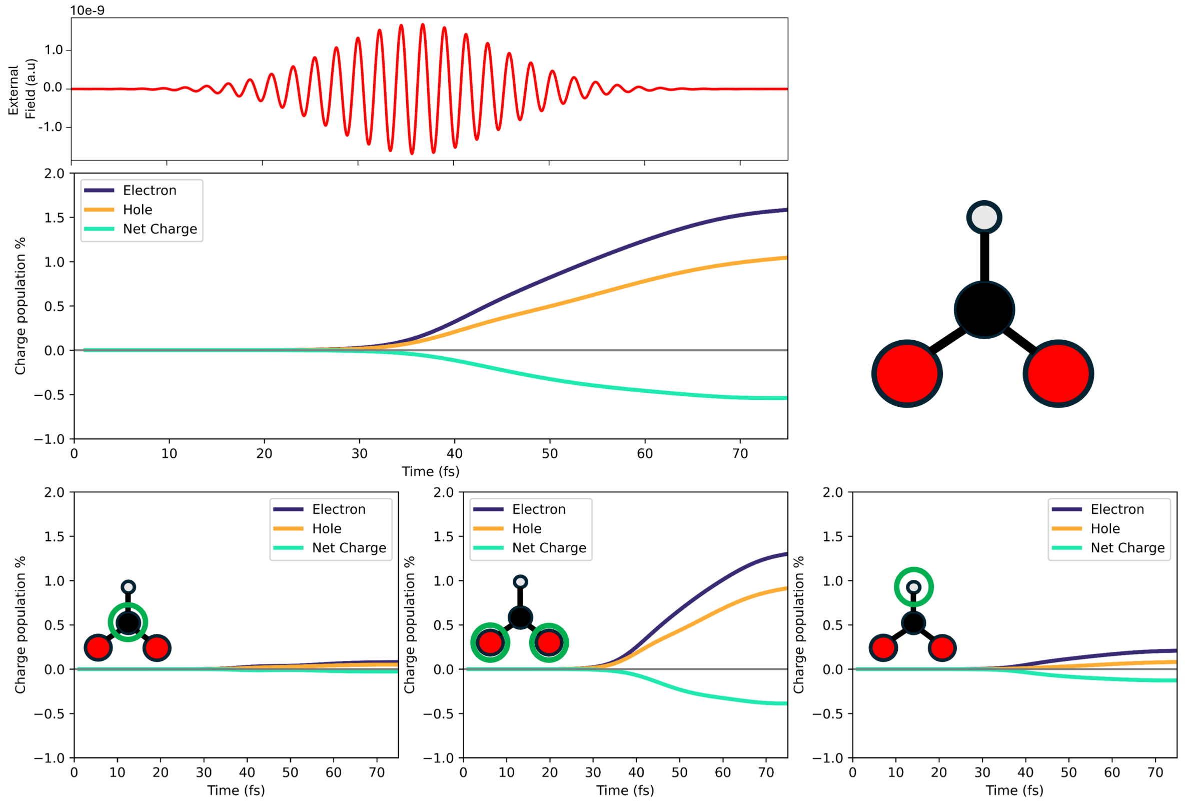

Figure S9: Upper panels: external pulse and time evolution of the photoinduced charge populations (electron, hole and net) of the HCOO* fragment in presence of the NR at 2 nm with P1 pulse. Bottom panels: on the left, time evolution of the photoinduced charge populations (electron, hole and net) of the carbon atom; in the middle, the same for the oxygen atoms; on the right, the same for the hydrogen atom of HCOO*.

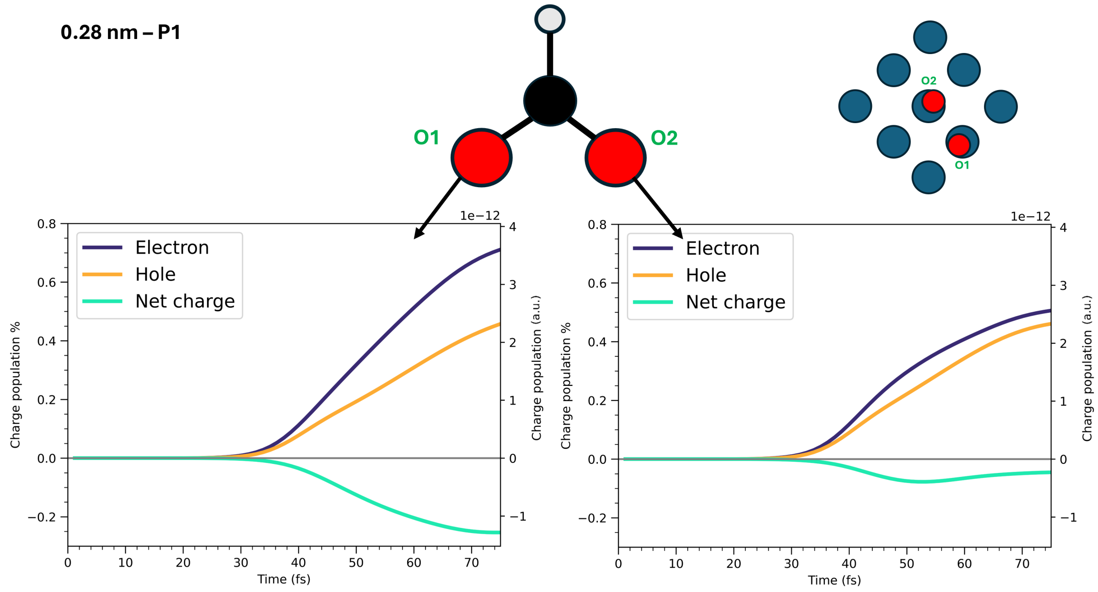

Figure S10: Time evolution of the photoinduced charge populations (electron, hole and net) of O1 and O2 atoms in the presence of the Au NR at 0.28 nm of distance with P1 pulse.

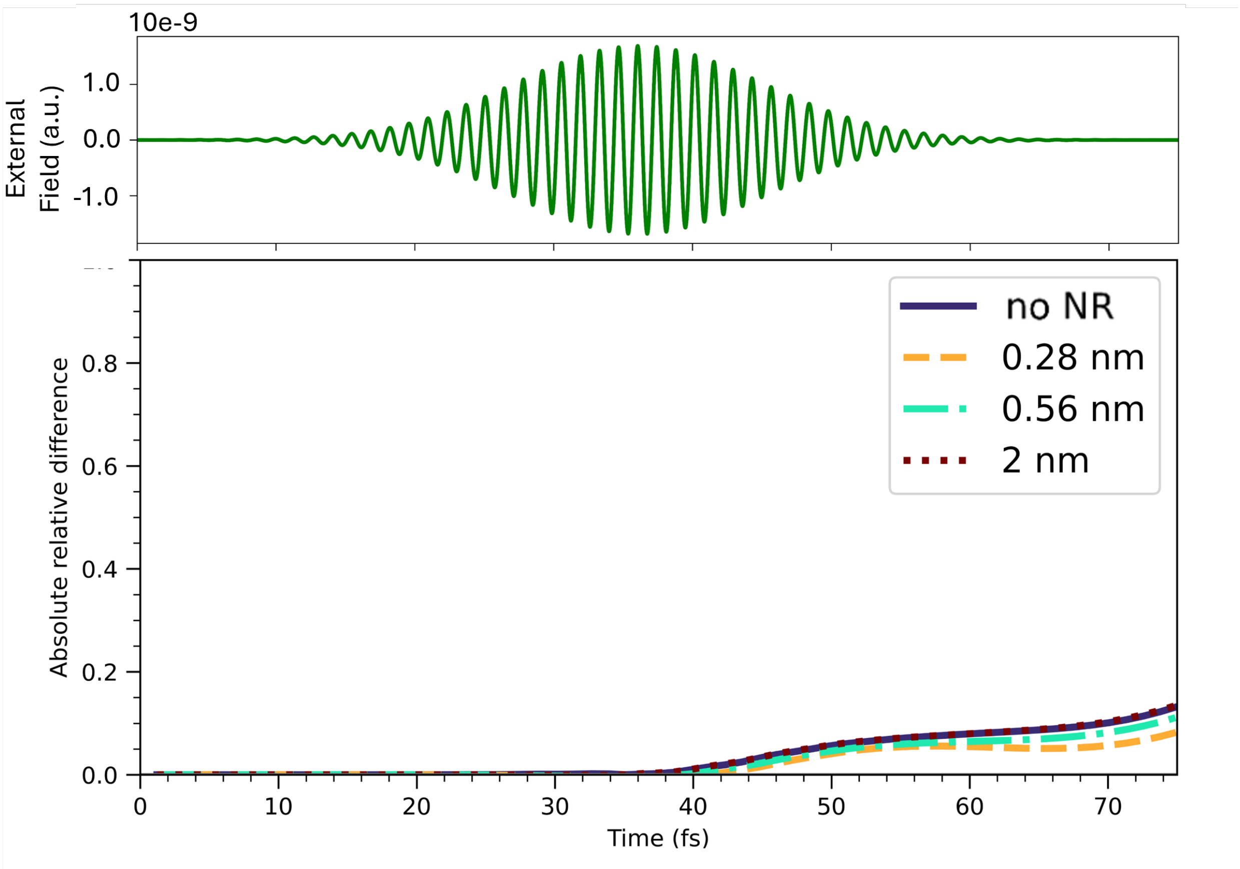

Figure S11: Absolute relative difference of the electron population of the two O atoms of HCOO* with the P2 pulse, at various distances between the QM subsystem and the NR. The case of the isolated QM subsystem is also reported for comparison. External pulse is also given.

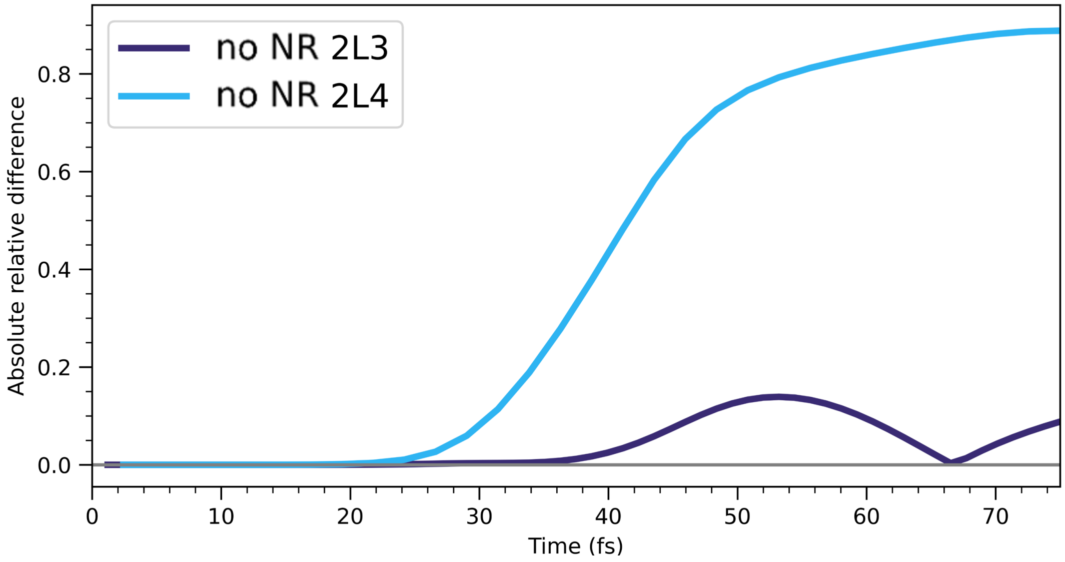

Figure S12: Absolute relative difference of the electron population of the two O atoms of HCOO* with the P1 pulse, for 2L3 and 2L4, without NR.

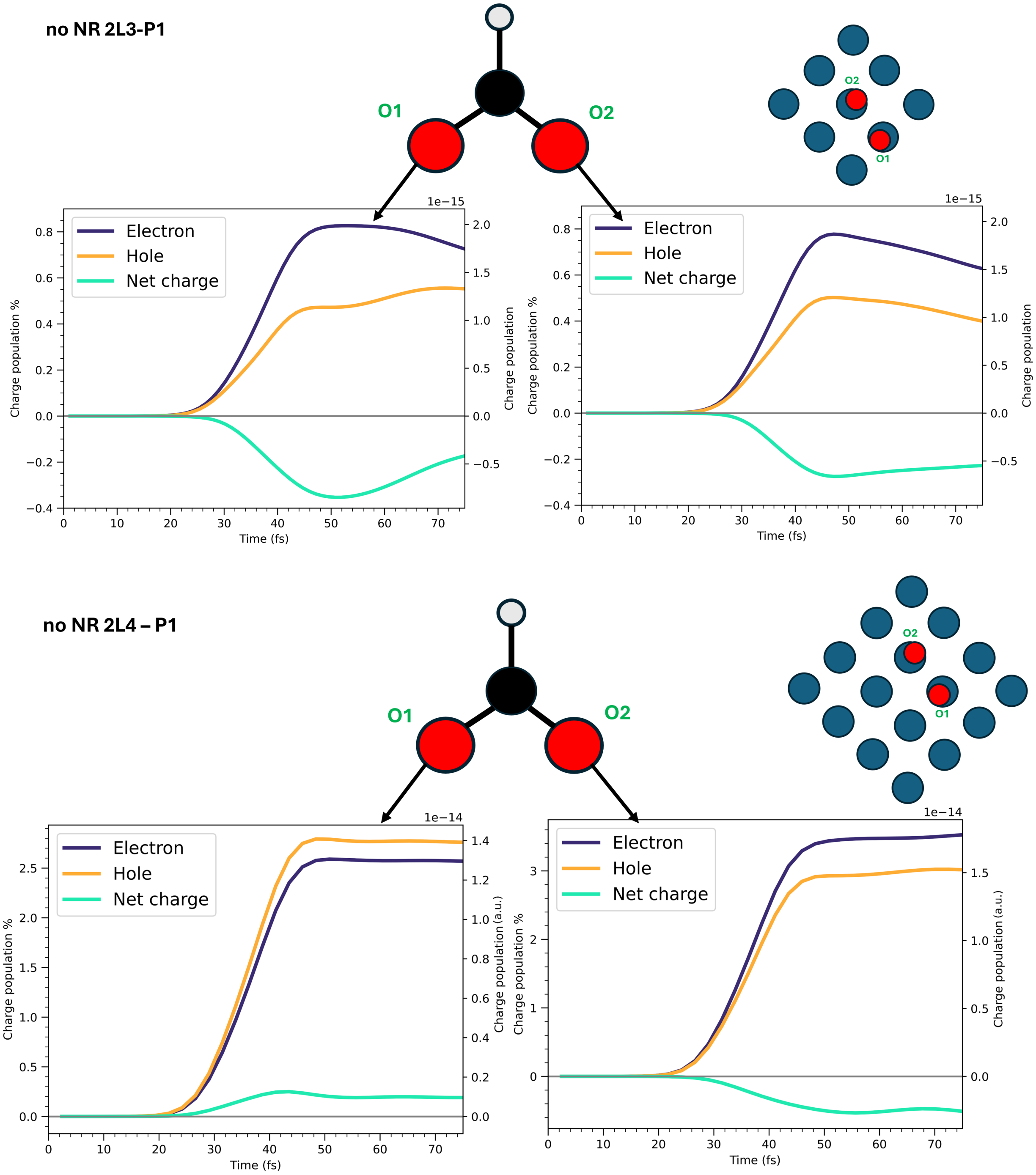

Figure S13: Top: Time evolution of the photoinduced charge populations (electron, hole and net) of O1 and O2 atoms in the bare 2L3 cluster (no NR) with P1 pulse. Bottom: Time evolution of the photoinduced charge populations (electron, hole and net) of O1 and O2 atoms in the bare 2L4 cluster (no NR) with P1 pulse.

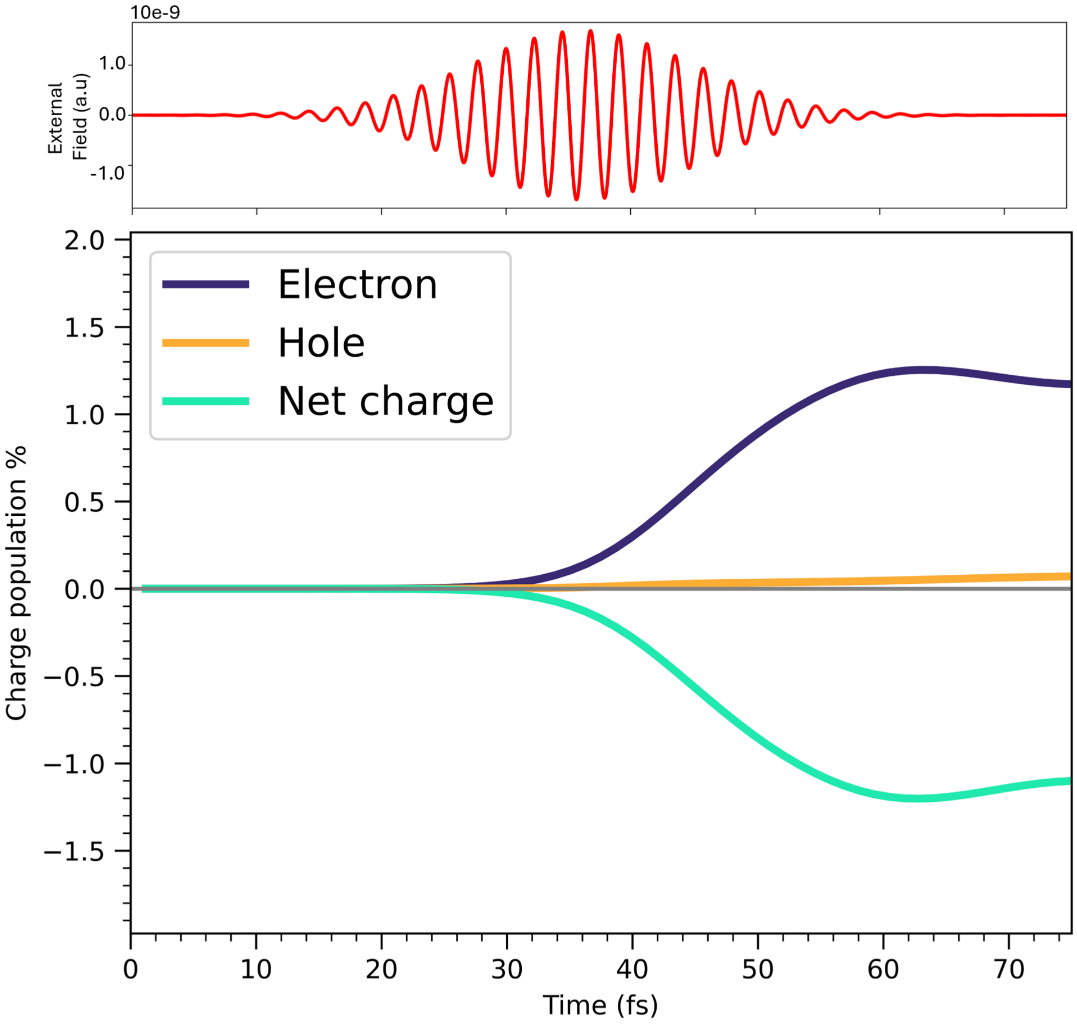

Figure S14: Time evolution of the photoinduced charge populations (electron, hole and net) of the H* fragment in presence of the NR at 0.28 nm with P1 pulse.

References

(1) Pipolo, S.; Corni, S. Real-Time Description of the Electronic Dynamics for a Molecule Close to a Plasmonic Nanoparticle. *J. Phys. Chem. C* **2016**, *120*, 28774.

(2) Dall'Osto, G.; Coccia, E.; Guido, C. A.; Corni, S. Investigating ultrafast two-pulse experiments on single DNQDI fluorophores: a stochastic quantum approach. *Phys. Chem. Chem. Phys.* **2020**, *22*, 16734.

(3) Marsili, M.; Corni, S. Electronic Dynamics of a Molecular System Coupled to a Plasmonic Nanoparticle Combining the Polarizable Continuum Model and Many-Body Perturbation Theory. *J. Phys. Chem. C* **2022**, *126*, 8768.

(4) Rüger, R.; van Lenthe, E.; Heine, T.; Visscher, L. Tight-Binding Approximations to Time-Dependent Density Functional Theory - a fast approach for the calculation of electronically excited states. *J. Chem. Phys.* **2016**, *144*, 184103.

(5) Hoerner, P.; Lee, M. K.; Schlegel, H. B. Angular dependence of strong field ionization of N_2 by time-dependent configuration interaction using density functional theory and the Tamm-Dancoff approximation. *J. Chem. Phys.* **2019**, *151*.

(6) Rüger, R.; Franchini, M.; Trnka, T.; Yakovlev, A.; van Lenthe, E.; Philipsen, P.; van Vuren, T.; Klumpers, B.; Soini, T. AMS 2025.1, SCM, Theoretical Chemistry, Vrije Universiteit, Amsterdam, The Netherlands. 2025; `http://www.scm.com`.

(7) Grobas Illobre, P.; Marsili, M.; Corni, S.; Stener, M.; Toffoli, D.; Coccia, E. Time-Resolved Excited-State Analysis of Molecular Electron Dynamics by TDDFT and Bethe–Salpeter Equation Formalisms. *J. Chem. Theory Comput.* **2021**, *17*, 6314.

(8) Dall'Osto, G.; Marsili, M.; Vanzan, M.; Toffoli, D.; Stener, M.; Corni, S.; Coccia, E. Peeking into the Femtosecond Hot-Carrier Dynamics Reveals Unexpected Mechanisms in Plasmonic Photocatalysis. *J. Am. Chem. Soc.* **2024**, *146*, 2208.

(9) Dall'Osto, G.; Vanzan, M.; Corni, S.; Coccia, M. M. E. Stochastic Schrödinger equation for hot-carrier dynamics in plasmonic systems. *J. Chem. Phys.* **2024**, *161*, 124103.

(10) Biancorosso, L.; Coccia, E. Study of the Photoinduced Charge Injection in the Reaction Intermediate of the Dehydrogenation of Formic Acid on Palladium. *J. Comput. Chem.* **2025**, *46*, e70087.

(11) Coccia, E.; Fregoni, J.; Guido, C. A.; Marsili, M.; Corni, S. Hybrid theoretical models for molecular nanoplasmonics. *J. Chem. Phys.* **2020**, *153*, 200901.

(12) Monti, M.; Stener, M.; Coccia, E. Electronic circular dichroism from real-time propagation in state space. *J. Chem. Phys* **2023**, *158*, 084102.

(13) Biancorosso, L.; D'Antoni, P.; Corni, S.; Stener, M.; Coccia, E. Time-dependent quantum/continuum modeling of plasmon-enhanced electronic circular dichroism. *J. Chem. Phys.* **2024**, *161*, 214104.

(14) Hammer, B.; Hansen, L. B.; Nørskov, J. K. Improved adsorption energetics within density-functional theory using revised Perdew-Burke-Ernzerhof functionals. *Phys. Rev. B* **1999**, *59*, 7413.
