# Supplementary material for: Plasmon-Enhanced Asymmetry in the Charge Distribution Explains the Increased H2 Production Rate from Formic Acid with a Pd-Tipped Au Nanorod
Source: J Phys Chem Lett. 2025 Dec 11;16(50):12931–8. doi: 10.1021/acs.jpclett.5c02858 (PMC12720282; doi:10.1021/acs.jpclett.5c02858)
Supplement: Supplementary file 2 [file jz5c02858_si_002.pdf]

jz-2025-028589.R1

Name: Peer Review Information for "Plasmon-enhanced asymmetry in the charge distribution explains the increased H₂ production rate from formic acid with a Pd-tipped Au nanorod"

First Round of Reviewer Comments

Reviewer: 1

Comments to the Author

In their paper, the authors present modelling results for the light-induced charge transfer from a Pd catalyst to adsorbed formic acid and how this quantity is affected by the presence of Au plasmonic nanorod underneath the Pd. They find that the presence of the nanorod significantly enhances charge transfer into the adsorbates. Moreover, they find different amounts of charge transfer into the two oxygen atoms which can facilitate the transformation from a bidentate to a monodentate adsorption. The authors state that this explains the results of experimental studies which have found an increase in H2 production on Pd-tipped gold nanorods.

Overall, I find the paper interesting. The results are well presented and discussed. Before I can make a recommendation regarding publication, however, I have several questions that the authors should address:

+ I am surprised that there is no backtransfer of charge from the adsorbate to the Pd. After the pulse has been switched off, the system should rapidly return to its ground state – however, this does not seem to happen in the simulations, see eg the charge populations in Fig. 2. If the backtransfer of charge is fast, then the nuclei do not have sufficient time to move and the reaction should not be affected by the phenomenon.

+ the nature and magnitude of light-induced charge transfer depends sensitively on the alignment of the adsorbate energy levels with the Pd energy levels. DFT often fails to provide accurate energy level alignments. Is there any evidence that the computational method used in this paper produces an accurate alignment of energy levels? Is there any confirmation from experiment that the magnitude and sign of the energy transfer is correct?

+ I am very surprised about the small magnitude of the net charge that is transfer; eg in Fig. 3 the transferred net charge is less than 10^-11 (unfortunately, the authors do not give any units for the charge on their graphs). I would have expected that approximately 1 electron gets transferred if one photon is absorbed.

+ small issues: some x-axis labels are very small (in particular those of the graphs that show the perturbing potential) and I would replace "NO NR" by "no NR", ie. Change the capitalization of the word "no".

+ It would be useful to explain more clearly the experimental measurement and which observations can be quantitatively or qualitatively explained by the simulation.

+ can the authors estimate the effect of charge transfer on the transition state barrier?

+ is the bilayer model of Pd realistic? How many Pd layers are used in the experiment?

Reviewer: 2

Comments to the Author

The manuscript addresses a timely and important question in plasmon-assisted catalysis: how microscopic mechanisms connect hot-carrier dynamics with catalytic activity. The

authors employ a multiscale computational framework, combining a quantum mechanical description of Pd with adsorbed intermediates (HCOO*, H*) and a classical plasmonic model of Au nanorods. Their simulations reveal that resonance excitation induces asymmetric charge injection into the oxygen atoms of formate, providing a microscopic explanation for the experimentally observed enhancement in H_2 production. In addition, the study highlights increased surface-charge heterogeneity on Pd under plasmon resonance, further clarifying the origins of the catalytic activity boost. I suggest publications once the following points are addressed/corrected:

1) The fixed-nuclei approximation is a limitation of the model: bond rearrangements (HCOO* → monodentate → H_2) are assumed rather than dynamically simulated. While this is acknowledged, further justification or discussion of the impact on results would strengthen the conclusions.

2) How this asymmetry in the O-Pd bonds (bond distances are not given but I supposed they are not identical) affects the asymmetry of the charge injection. This point need to be discussed.

3) Figure S12 shows the asymmetry in the charge injection as a function of the Pd cluster size. There is a huge effect that is not completely clear to me. Can the author clarify this point?

Minors points:

Figures S10–S12 (SI): The labels "inner" and "outer O atom" could be clarified with a structural diagram.

Typos

Page 2: "Herran and et al." → should be "Herran et al."

Page 7: "electron, hole and hole" in Figure 5 caption seems to be a typo.

Author's Response to Peer Review Comments:

Plasmon-enhanced asymmetry in the charge distribution explains the increased H_2 production rate from formic acid with a Pd-tipped Au nanorod

Leonardo Biancorosso and Emanuele Coccia*

Dipartimento di Scienze Chimiche e Farmaceutiche, University of Trieste, via L. Giorgieri 1, 34127, Trieste, Italy

E-mail: ecoccia@units.it

We would like to thank the two reviewers for their careful reading and analysis of our work. Their comments have enabled us to improve the quality of the new manuscript, correcting typos, strengthening conclusions, and linking our results more directly to the experimental data. We provide point-by-point responses below, with all changes in the revised text highlighted in red.

Reviewer 1

In their paper, the authors present modelling results for the light-induced charge transfer from a Pd catalyst to adsorbed formic acid and how this quantity is affected by the presence of Au plasmonic nanorod underneath the Pd. They find that the presence of the nanorod significantly enhances charge transfer into the adsorbates. Moreover, they find different amounts of charge transfer into the two oxygen atoms which can facilitate the transformation from a bidentate to a monodentate adsorption. The authors state that this explains the results of experimental studies which have found an increase in H2 production on Pd-tipped gold nanorods.

Overall, I find the paper interesting. The results are well presented and discussed. Before I can make a recommendation regarding publication, however, I have several questions that the authors should address:

+ I am surprised that there is no backtransfer of charge from the adsorbate to the Pd. After the pulse has been switched off, the system should rapidly return to its ground state – however, this does not seem to happen in the simulations, see eg the charge populations in Fig. 2. If the backtransfer of charge is fast, then the nuclei do not have sufficient time to move and the reaction should not be affected by the phenomenon.

Simulations have been carried out for a closed system, i.e. the electron dynamics is coherent. When the pulse is switched off, no other perturbation affects the electronic degrees of freedom. Thus, a charge backtransfer is not included in the reported simulations. In our J. Chem. Phys. 161, 124103 (2024) on another photocatalytic system (Ref. [29] in the revised manuscript), we use the theory of open quantum systems to account for relaxation channels, which indeed make the charge injection being transient.

We thank the reviewer for arising this point, which was not discussed in the first version of the work. We have added a comment on page 4: "All the simulations refer to a closed system according to which electron dynamics is coherent. When the pulse is switched off, no other perturbation affects the electronic degrees of freedom. Effective approaches to account for charge relaxation and backtransfer could be used [29]."

+ the nature and magnitude of light-induced charge transfer depends sensitively on the alignment of the adsorbate energy levels with the Pd energy levels. DFT often fails to provide accurate energy level alignments. Is there any evidence that the computational method used in this paper produces an accurate alignment of energy levels? Is there any confirmation from experiment that the magnitude and sign of the energy transfer is correct?

We agree with the reviewer that having an accurate energy-level alignment is crucial. Even though DFT is the most widely used approach, thanks to its favorable scaling and "black-box" nature, we also agree that it can fail in getting the right density of states. Alternative options are provided by wavefunction methods or GW/BSE.

In our recent work (Ref. [38] in the revised draft), we have shown that B3LYP provides the same physical information, i.e. an electron injection into HCOO*, thus providing additional control over the reliability of our current results. We have added this comment on page 4: ”In our recent work [38], we have shown that B3LYP provides the same physical information, i.e. an electron injection into HCOO*, thus providing additional control over the reliability of the current results. Using more refined approaches such as GW/BSE would be computationally too demanding, since 1808 electronic states have been computed and then propagated in this work.”

On the other hand, we must always find a balance between accuracy and computational efficiency. In fact, as reported in the Supporting Information, we calculated 1808 electronic states with AMS, which were then propagated with WaveT. Working with such a large size at the GW/BSE or CASSCF level is probably too computationally burdensome.

Moreover, authors of the experimental give evidence of an electron transfer from Au to Pd, in terms of a strong quenching of emission at the LSPR region. They also state that this charge transfer and the subsequent charge redistribution enhance the interaction between the adsorbate and the Pd surface. However, no microscopic detail on how this enhanced catalytic activity is obtained , i.e. the type of charge injection into the adsorbate, is provided.

+ I am very surprised about the small magnitude of the net charge that is transfer; eg in Fig. 3 the transferred net charge is less than 10^{-11} (unfortunately, the authors do not give any units for the charge on their graphs). I would have expected that approximately 1 electron gets transferred if one photon is absorbed.

The reviewer is right about the units of the charge. They are atomic units, which have been added in the revised figures. The amount of charge transferred depends on the pulse intensity, which is in our simulations is low enough to guarantee that only a linear response is considered. We have added the following comment on page 4 of the revised manuscript: ” The pulse peak intensity is equal to 10^2W/cm^2 and a Gaussian envelope with a full width at half maximum (FWHM) of 21 fs has been adopted, See Eq. 6 of SI. This FWHM was chosen to approximate the effect of continuous-wave light with a coherence length of a few microns, consistent with experimental conditions [10]. The photoresponse is therefore in the linear regime.”

Small values of photocharge are consistent with a low incident photon conversion efficiency, with values between 2 and 5% at the plasmon resonance peak, as discussed in our Ref. [10].

+ small issues: some x-axis labels are very small (in particular those of the graphs that show the perturbing potential) and I would replace “NO NR” by “no NR”, ie. Change the capitalization of the word “no”.

We have modified the mentioned labels and legend notations according to the reviewer’s suggestion.

+ It would be useful to explain more clearly the experimental measurement and which observations can be quantitatively or qualitatively explained by the simulation.

We agree with the reviewer that a clearer connection with the experimental findings is needed.

We have added the following comment on page 3 of the new draft: ”The key experimental quantity is the H_2 production rate, which is enhanced in presence of the gold NR and maximized at the NR plasmon frequency. In Ref. [13], the authors focus on the plasmonic near field and on hot electrons to explain the observation. The idea behind our work is to complete the analysis, providing a direct link between the observed increase in H_2 production and the plasmonic effects that modify the charge injection in the bidentate HCOO*, thus making the reaction more efficient towards the products.”

+ can the authors estimate the effect of charge transfer on the transition state barrier?

EC: In order to answer the reviewer’s question, we have explored different strategies: i) optimizing the geometry of the bidentate adsorbed species and that of the monodentate one with an extra negative charge, in presence of Pd atoms: ii) optimizing the two negatively charge structures in the excited state corresponding to the plasmon frequency. In both case, the idea was to mimic the electron injection observed in our dynamics, getting structural information and possibly using the two optimized geometries to run a minimum-energy path calculation to estimate the activation energy. Unfortunately, we were unsuccessful with any of the strategies due to the high instability of the SCF step and the optimization of the geometry itself, which never converged. Therefore, we cannot respond positively to the reviewer’s request by providing him/her with an estimate of the change in the activation barrier for this step of the reaction.

+ is the bilayer model of Pd realistic? How many Pd layers are used in the experiment?

A 2-nm Pd layer is observed experimentally, deposited on the NR. Such a size can not be described with a quantum treatment within our computational strategy. However, we have confirmed electron injection as a function of the number of Pd atoms and their connection,

by studying 2- versus 3-layer Pd cluster in our recent work (Ref. [38]). While this is not yet conclusive proof of the reliability of the reactor model, it does allow us to consider our results as reliable. We have added the following comment on pages 3/4 of the revised manuscript: ”Even though our quantum model of the reactor is far from the experimental size, the Pd layer being 2-nm thick as shown in Ref. [38], our recent study on the shape and size of Pd cluster allows us to consider the present results, specifically the sign of charge injection, as robust.”

Reviewer 2

The manuscript addresses a timely and important question in plasmon-assisted catalysis: how microscopic mechanisms connect hot-carrier dynamics with catalytic activity. The authors employ a multiscale computational framework, combining a quantum mechanical description of Pd with adsorbed intermediates (HCOO*, H*) and a classical plasmonic model of Au nanorods. Their simulations reveal that resonance excitation induces asymmetric charge injection into the oxygen atoms of formate, providing a microscopic explanation for the experimentally observed enhancement in H production. In addition, the study highlights increased surface-charge heterogeneity on Pd under plasmon resonance, further clarifying the origins of the catalytic activity boost. I suggest publications once the following points are addressed/corrected:

1) The fixed-nuclei approximation is a limitation of the model: bond rearrangements (HCOO* → monodentate → H_2) are assumed rather than dynamically simulated. While this is acknowledged, further justification or discussion of the impact on results would strengthen the conclusions.

We have added the following comment on page 13: "Since the plasmonic field decays in 10/20 fs, we have assumed that nuclear motion is decoupled from the electron dynamics occurring in this time window, assuming the reaction pathway from literature. Though freezing nuclei can be a crude assumption, however, we believe that reliable information about the plasmonic role in modifying reaction steps can be provided by such a modelling."

2) How this asymmetry in the O-Pd bonds (bond distances are not given but I supposed they are not identical) affects the asymmetry of the charge injection. This point need to be discussed.

EC: We thank the reviewer for clarifying this point, that is indeed essential to enforce our conclusions. We have added this comment on pages 10/11 of the revised main text: "Distances are 2.12 (2.18) ˚Afor the O1 (O2). Asymmetry is inherent in the system, as can be seen from the result without NR: an asymmetrical charge population is present due to the adsorption of HCOO* on the Pd surface. We emphasize that, as described in detail in

the SI, the geometry optimization was conducted in periodic boundary conditions, starting from literature data. The presence of NR increases the asymmetry, thus leading more quickly to the monodentate species and to the final product, i.e., H_2."

3) Figure S12 shows the asymmetry in the charge injection as a function of the Pd cluster size. There is a huge effect that is not completely clear to me. Can the author clarify this point?

We agree with the reviewer about a needed comment to clarify the findings in Figure S12, which reports the asymmetry in oxygen charge population for the two bare, i.e. no NR, 2L3 and 2L4 systems. Indeed, a TD-PCM-NP dynamics with 2L4 would have been computationally much more demanding. To explain the difference between 2L3 and 2L4, we have added the new Figure S13 of Supporting Information, which contains the electron, hole and net charge populations for the two oxygen atoms in bare 2L3 (upper panels) and 2L4 (lower panels). For 2L4 we observe that the two atoms are characterized by a net charge of opposite sign, with the electron one being larger in absolute value. This result depends on the shape of the molecular orbitals involved in the dynamics, which differ from those in the 2L3 case, where both oxygen atoms are negatively charged and border effects play a nonneglible role. Therefore, a larger asymmetry is found for 2L4. We emphasize that this comparison only tells us that even in the most unfavorable case, where we use the smallest reactor model (2L3), the evidence of asymmetry is clear. Above all, we are interested in showing how plasmonic effects increase asymmetry in the charge population of oxygen atoms, rather than quantifying its value.

We have added the following comment on page 11: "Asymmetry in 2L4 is much larger than in 2L3. The reason of that is the two atoms being characterized by a net charge of opposite sign, with the electron one larger in absolute value (Figure S13 of SI). This result depends on the shape of the molecular orbitals involved in the dynamics, which differ from those in the 2L3 case, where both oxygen atoms are negatively charged and border effects play a nonneglible role."

Minors points:

Figures S10–S12 (SI): The labels "inner" and "outer O atom" could be clarified with a structural diagram.

We agree with the reviewer. We added in the new Figure S10 a scheme which unequivocally defines the two O atoms for the 2L3 cluster (now labelled O1 and O2), and a short sentence on page 10. We have done the same for 2L4 in the new Figure S13.

Typos:

Page 2: ”Herran and et al.” → should be ”Herran et al.”

We thank the reviewer. We fixed the typo.

Page 7: ”electron, hole and hole” in Figure 5 caption seems to be a typo.

We thank the reviewer. We fixed the typo.
